# Concept and benefits of the Inverted Classroom method for a competency-based biochemistry course in the pre-clinical stage of a human medicine course of studies

**DOI:** 10.3205/zma001108

**Published:** 2017-08-15

**Authors:** Susanne J. Kühl, Matthias Toberer, Oliver Keis, Daniel Tolks, Martin R. Fischer, Michael Kühl

**Affiliations:** 1 Ulm University, Institute of Biochemistry and Molecular Biology, Ulm, Germany; 2 Ulm University, Medical Faculty Ulm, Office of Student Affairs, Ulm, Germany; 3 Clinic of the Ludwig-Maximilians-University Munich, Institute of Didactics and Educational Research in Medicine, Munich, Germany

**Keywords:** Inverted Classroom, E-Learning, Biochemistry, human medicine, competency-based training, Masterplan Medizin 2020, NKLM

## Abstract

**Background: **Medical students often have a problem recognising the relevance of basic science subjects for their later professional work in the pre-clinical stage of their studies. This can lead to a lower motivation to learn biochemical content and dissatisfaction in the courses amongst the students. Alternative teaching methods such as the Inverted Classroom (IC) method can address this deficiency. The goal of this study was:

1. to analyse the motivation and satisfaction of the students in a biochemistry seminar through the use of the e-learning-based IC method,
2. to investigate the acceptance against the IC teaching method in biochemistry, and
3. to compare the learning success achieved using the IC approach with that of a traditional course.

We also investigated how a biochemistry course in the pre-clinical stage of a human medicine course of studies can be successfully organised according to the IC method. Furthermore, we examined the benefits of the IC method over conventional teaching formats.

**Method: **The IC method was implemented in accordance with the guidelines of the GMA committee “New Media” [30] in a biochemistry seminar for two student IC intervention groups with 42 students. A part of the factual knowledge from the on-site phase in the form of teaching videos together with self-learning control tasks were provided online before the seminar for both IC intervention groups. Exporting content to the self-learning phase creates new free time in the on-site phase, during which the content can be critically considered and processed and additional competency-based learning objectives can be taught. Identical biochemistry teaching content was taught in parallel control groups (14 student groups with n=299 students), but no material was handed out beforehand for a self-learning phase. These students only received the materials after the on-site phase. Motivation and satisfaction as well as the acceptance for the teaching methods were recorded by questionnaires, the acquisition of knowledge by MC exams.

**Results:** On a Likert scale from 1 (strongly disagree) to 6 (strongly agree), the students in the IC intervention groups could be seen to be much more motivated (5.53) than students in the control group (4.01). Students in the IC intervention groups also recognised the relevance of the learning content much more clearly (5.44) than students in the control group (4.01). Furthermore, the IC group also observed that additional competencies were trained in addition to the biochemistry content. In addition, the IC intervention group award the event a school grade of 1.53, the traditional control group a grade of 2.96. The teaching videos were rated very positively by both groups with an average school grade of 1.3 in each case. A qualitative analysis showed that the motivation and a positive attitude of the lecturers played a decisive role in the successful implementation of the IC method.

**Discussion and conclusion: **Pre-clinical students display a high acceptance of the e-learning-based IC method. Teaching communication competencies in a biochemistry seminar was also rated very positively by the students. The quality of the teaching video and the motivation of the lecturers were shown to be a critical parameter for the successful performance of the IC method. What’s more, the IC method can contribute to implementing a competence orientation in medical studies.

## Zusammenfassung

**Hintergrund: **Häufig haben Medizinstudierende Probleme, die Relevanz grundlagenwissenschaftlicher Fächer für die spätere berufliche Tätigkeit bereits im vorklinischen Studienabschnitt zu erkennen. Dies kann bei den Studierenden zu einer geringen Motivation für das Erlernen biochemischer Inhalte sowie zur Unzufriedenheit in Lehrveranstaltungen führen. Alternative Lehrmethoden wie die Inverted Classroom (IC)-Methode könnten diesem Mangel begegnen. Ziel dieser Studie war es,

1. die Motivation und Zufriedenheit der Studierenden in einem Biochemie-Seminar durch den Einsatz der E-Learning unterstützten IC-Methode zu analysieren,
2. die Akzeptanz gegenüber der IC-Lehrmethode im Fach Biochemie zu untersuchen und
3. den durch den IC-Ansatz erzielten Lernerfolg mit dem einer traditionellen Lehrveranstaltung zu vergleichen.

Zusätzlich wurde untersucht, wie sich eine Biochemie Lehrveranstaltung im vorklinischen Studienabschnitt der Humanmedizin erfolgreich nach der IC-Methode durchführen lässt und welchen Nutzen die IC-Methode gegenüber herkömmlichen Lehrformaten hat.

**Methode:** Die IC-Methode wurde entsprechend der Leitlinien des GMA-Ausschusses „Neue Medien“ [30] in einem Biochemie-Seminar für zwei studentische IC-Interventionsgruppen mit 42 Studierenden implementiert. Für die beiden IC-Interventionsgruppen wurde ein Teil des Faktenwissens aus der Präsenzphase in Form von Lehrvideos zusammen mit Selbstlernkontrollaufgaben vor dem Seminar online zur Verfügung gestellt. Durch das Auslagern von Inhalten in die Selbstlernphase ergaben sich neue zeitliche Freiräume in der Präsenzphase, in denen die Inhalte kritisch reflektiert und verarbeitet und zusätzliche kompetenzorientierte Lernziele vermittelt wurden. In parallelen Kontrollgruppen (14 studentische Gruppen mit n=299 Studierenden) wurden identische biochemische Lehrinhalte vermittelt, jedoch vorab keine Materialien für eine Selbstlernphase ausgehändigt. Diese Studierenden erhielten die Materialien erst nach der Präsenzphase. Motivation und Zufriedenheit sowie die Akzeptanz für die Lehrmethoden wurde durch Fragebögen, der Wissenserwerb durch MC-Klausuren erhoben.

**Ergebnisse: **Auf einer Likert-Skala von 1 (trifft gar nicht zu) bis 6 (trifft vollkommen zu) zeigten sich Studierende der IC-Interventionsgruppen mit 5,53 signifikant motivierter im Vergleich zu Studierenden der Kontrollgruppe mit 4,01. Auch erkannten Studierende der IC-Interventionsgruppen mit 5,44 die Relevanz der Lerninhalte signifikant deutlicher als Studierende der Kontrollgruppe mit 4,01. Weiterhin nahmen die IC-Gruppen wahr, dass neben den biochemischen Inhalten zusätzliche Kompetenzen trainiert wurden. Darüber hinaus vergab die IC-Interventionsgruppe der Veranstaltung eine Schulnote von 1,53, die traditionelle Kontrollgruppe eine Note von 2,96. Die Lehrvideos wurden von beiden Gruppen mit einer Durchschnittsschulnote von je 1,3 sehr positiv bewertet. Eine qualitative Auswertung ergab, dass Motivation und positive Einstellung der Dozierenden eine entscheidende Rolle für die erfolgreiche Umsetzung der IC-Methode spielte.

**Diskussion und Schlussfolgerung:** Studierende der Vorklinik zeigen eine hohe Akzeptanz gegenüber der E-Learning unterstützten IC-Methode. Weiterhin wurde die Vermittlung von Kommunikationskompetenzen im Biochemie-Seminar von den Studierenden sehr positiv bewertet. Als kritische Parameter für die erfolgreiche Durchführung der IC-Methode zeigten sich die Qualität der Lernvideos und die Motivation der Lehrenden. Darüber hinaus kann die IC-Methode dazu beitragen, eine Kompetenzorientierung im Medizinstudium zu implementieren.

## 1. Introduction

### 1.1. Definition of the problem

Human medicine students often have a problem recognising the medical relevance of basic science subjects such as biochemistry in the pre-clinical stage of their studies for their degree course in general as well as their later professional work (own observations and discussions with other lecturers from the pre-clinical phase). As a result, the motivation to come to terms with the learning content is often very low, leading to an unsatisfactory learning atmosphere in the courses. Moreover, the instruction is often limited to simply learning facts and is neither student-friendly nor competency-based.

The “integrated seminar pursuant to § 2 (2) ÄAppO (German Medical License Act)“ is a compulsory course for all students of human medicine in the pre-clinical stage of their studies. The Institute of Biochemistry and Molecular Biology of Ulm University is hereby responsible for one of seven subject areas with the topic “Pathobiochemistry II: From gene to protein“, that takes place in the second semester. 16 groups of around 20 students each are taught every semester by one lecturer each. The on-site phase hereby covers two appointments of four clock hours each.

The analysis of the official evaluation sheets from the Medical Faculty from the year 2015 showed that a large number of students were not satisfied with the seminar and that there was a need for optimisation in various areas. For example, around 25% of students saw a need for optimisation in the field of methodology and didactics. This deficiency could be addressed by introducing a new teaching concept. One possible teaching concept here is the Inverted Classroom (IC) method [15], which initial studies have in the meantime classified as being conducive to learning [1], [20]. The IC method has already been put to successful use in medical studies [3], [11], [19], [25], [29], [31], as well as dentistry [2], pharmaceutics [6], [17], [22], nursing [5], [18] and other healthcare professions [7], [16]. The method is described by some leading scientists and institutions as a sensible approach to teaching and training in healthcare professions [23], [32], a Horizon Report 2014 even calls it one of the most important developments in teaching and learning technology [13]. With this method, a certain subject matter is initially appropriated in a self-learning phase. This matter is then applied in the following on-site phase, transferring the passive into active knowledge so that a higher level of learning can be achieved. Various studies have already proven that students’ motivation can be increased by the use of the IC method [9]. A study from the field of biochemistry in particular shows that students were more satisfied with the transfer of biochemical knowledge by means of the IC method than with traditional teaching methods [21], [23]. A publication by the “New Media” committee of the GMA has already drawn up guidelines for the use of the IC method in human medicine studies [30].

The evaluation sheets of 2015 also showed that around 30% of students saw a need for optimisation with respect to the seminar content because they were unable to recognise the medical relevance of the teaching content. This is in line with the recently published Masterplan Medizin 2020, which includes, amongst other things, an early integration of clinical matters and a stronger emphasis on competence-based training in medical studies [https://www.bmbf.de/de/masterplan-medizinstudium-2020-4024.html]. The use of the IC method creates more free time in the on-site phase, during which additional communicative competencies can be taught on the basis of clinical-scientific cases. This free time also allows the use of further didactic methods that encourage the active cooperation of the students to enable a stronger transfer of passive to active knowledge. In addition, the content and the accompanying medical relevance was to be made transparent by a definition of clear learning objectives according to the National Competence Based Catalogue of Learning Objectives for Medical Education (NKLM, Nationaler kompetenzbasierter Lernzielkatalaog Medizin) [http://www.nklm.de]. 

However, there is as yet very little knowledge about the use and efficacy of the IC method in the pre-clinical stage of studies, including the subject biochemistry. This is why the IC method will be used in the aforementioned integrated seminar: “Pathobiochemistry II: From gene to protein” and its success measured in this study.

#### 1.2. Issues in this study

This exploratory field study dealt with the following issues:

1. Can the motivation and satisfaction of students of human medicine be increased through the use of the e-learning-based IC method in a biochemistry seminar in the second pre-clinical semester?
2. Would the IC method in the biochemistry seminar in the second pre-clinical semester in human medicine be accepted by the students?
3. Is there an improvement in the learning success through the use of the IC compared to the traditional teaching method in the biochemistry seminar in the second pre-clinical semester in human medicine?
4. How can a biochemistry course be planned and successfully organised according to the IC method in the second pre-clinical semester?
5. What is the added value of the IC method compared to conventional teaching methods in the second pre-clinical semester?

## 2. Methods and implementation

### 2.1. Participants in the study, classification and ethics

The participants in the study consisted of the cohort of students of human medicine in their second semester in the summer semester 2016 at Ulm University. The cohort consisted of a total of 341 students, though not all of the students took part in all voluntary evaluations of the test.

In Ulm, students are basically assigned to groups by the Office of Student Affairs of the Medical Faculty without any influence from the lecturers. This classification usually takes place at random, whereby a small number of students swap groups so that they can take part in elective subjects. Thus 14 groups with 299 students were assigned at random to the traditional control group and 2 groups with 42 students to the IC intervention group. All of the groups were taught by experienced lecturers (see below for more details). 

A total of 220 students took part in the evaluation using the general sheets of the medical faculty. These included 32 students from the IC intervention and 188 from the traditional control groups. 42 students took part in the special evaluation of the IC course, and 112 students in the evaluation of the teaching films (control group: n=72; IC group: n=40). A total of up to 42 students took part in the knowledge test within the IC group. The results of all participants in the subsequent biochemistry exam were evaluated (control group: n=299; IC group: n=40). 

The project was submitted to the ethics commission of Ulm University for assessment. An official application was not considered necessary. The anonymity of all data was guaranteed at all times. 

#### 2.2. Object of the study: Inverted Classroom versus traditional teaching concept

Two IC intervention groups with a total of 42 students were taught according to the e-learning-based IC method. These IC intervention groups were taught by an experienced biochemistry lecturer (S.J. K.) who implemented the IC method, including the teaching of competency-based learning objectives, for the first time. A further 14 control groups (n=299) were taught according to the original, traditional teaching concept. The traditional control groups were largely taught by experienced lecturers (incl. M.K.) who were familiar with the content and organisation of the seminar. 

**Figure 1:**
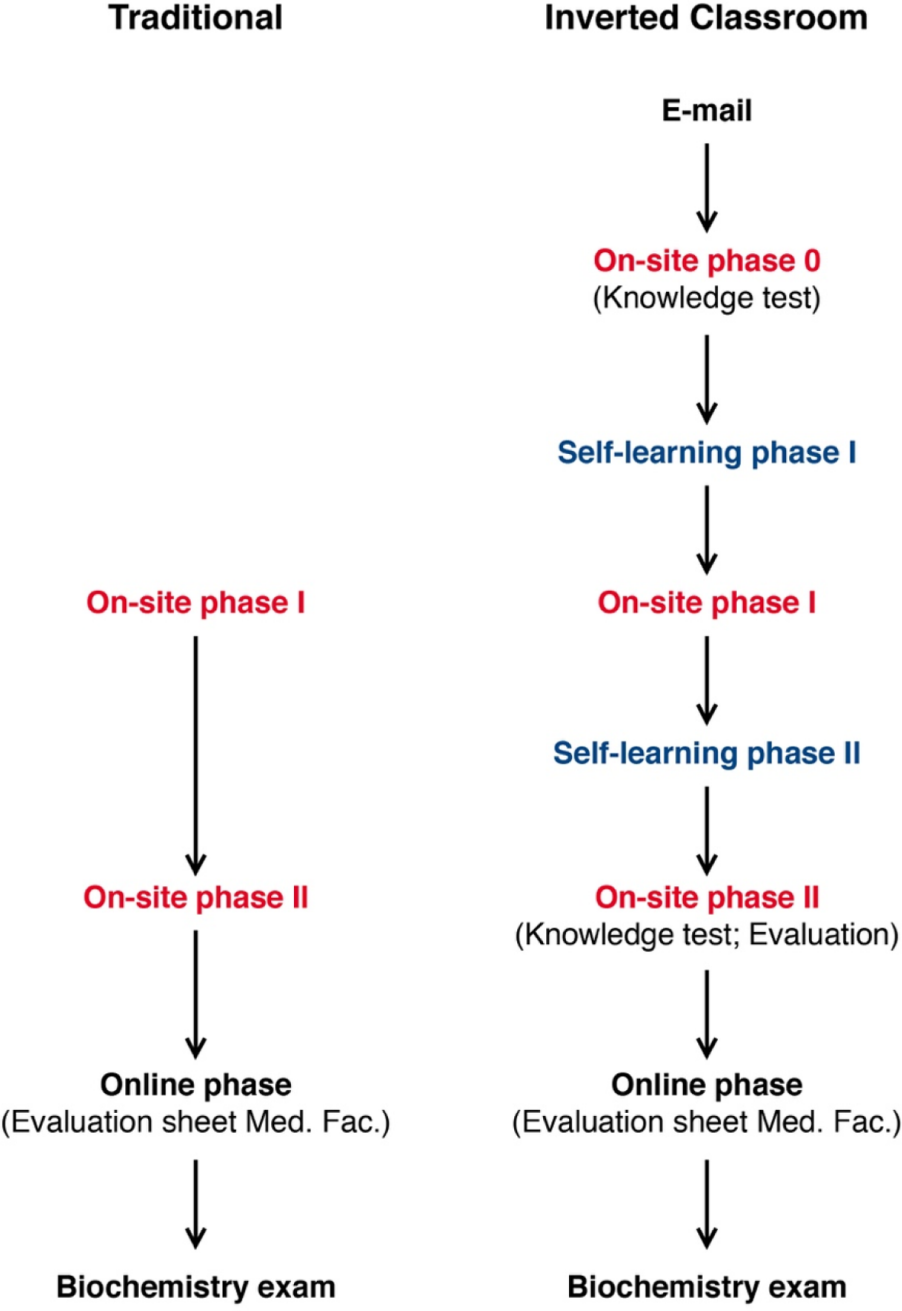
Comparison of the two teaching concepts Left: Traditional teaching concept. Two on-site phases of four full clock hours each were held. A clinical case with corresponding questions and tasks was handed out in paper form in each of the 2 four-hour courses. After an around one-hour preparatory phase, the tasks were addressed jointly, for example in discussion groups or by student presentations. Tuition was mainly in the form of lecturer-centred instruction. In a subsequent online phase the students received on the one hand the preparatory materials of the IC group (i.e. after the seminar), and on the other the official evaluation sheet of the Medical Faculty in Ulm. The official biochemistry exam was held at the end of the semester. The on-site phase 0 was not carried out for the traditional teaching concept because 1. the traditional conditions were not to be changed, and 2. this was not necessary since the students did not have to be prepared for a self-learning phase. Right: Inverted Classroom teaching concept. The course was initiated by an e-mail inviting the students in the IC group to an on-site phase 0 with a brief explanation. A 45-minute introductory event (on-site phase 0) thus took place 1.5 weeks before on-site phase I. During this event, the students were presented with last year’s evaluation results for the integrated seminar as well as the goals of the new conception of the course. The organisational procedure for the seminar was then explained. What’s more, a formative, written and anonymised knowledge test was held with MC questions and case-based questions (see Attachment 1, Knowledge test). This was followed by self-learning phase I. The students were given exact work instructions for the self-learning phase. The students were asked to study a teaching film including self-learning control tasks in self-learning phase I. The complete group were told to watch a documentary film on Osteogenesis imperfecta. On-site phase I lasted for a total of four full clock hours (see Attacment 1). Osteogenesis imperfecta (OI) was discussed as a clinical symptom. Communication in a team was also trained in a group work phase and communication in an oral exam with feedback in an exam simulation (please refer to the Attachment 1 for exemplary work assignments from on-site phases I and II) in on-site phase I. Self-learning phase II was carried out similar to self-learning phase I, except that the students were allowed to watch two teaching films and two documentary films about Ehlers-Danlos syndrome and scurvy. On-site phase II was carried out similar to on-site phase I, whereby this unit concentrated on communication with peers and laymen (see Attachment 1). The knowledge test was held again at the end of the seminar and the evaluation sheet handed out for the course and teaching videos. In a subsequent online phase the students received the official evaluation sheet of the Medical Faculty in Ulm. An e-mail survey was carried out with the IC group. The official biochemistry exam was held at the end of the semester.

A comparison of the seminar procedures according to the traditional and the IC teaching methods can be seen in Figure 1 (Fig. 1). Details of the procedure and the teaching content are shown in the Attachment 1. 

#### 2.3. Material for the self-learning phases

##### 2.3.1. Teaching films

All of the illustrations were generated using the Adobe Illustrator graphic design software. Three teaching films, each lasting around 10 minutes, were produced using the Keynote and Camtasia programs. The first teaching film taught the biochemical principles of protein biosynthesis. The second teaching film presented protein biosynthesis into the rough endoplasmatic reticulum and the subsequent vesicle transport. The third film dealt with the structure and formation of the collagen triple helix and fibrils. The optimised teaching films used in the seminar are available from the Institute of Biochemistry and Molecular Biology, Ulm University. Three comprehension questions were posed for each film as a self-learning control (see Attachment 1, self-learning tasks). 

##### 2.3.2. Learning objectives and course description

The course described here is a second semester seminar in which the content of the lectures in the first semester is consolidated and applied. The learning objectives cover theoretical and methodical content as well as clinical references in the form of exemplary medical conditions. The topics of amino acids, proteins, enzymes, topobiochemistry (cell biology) and molecular biology are dealt with. The learning objectives were placed at the disposal of all students in the first semester via the learning platform Moodle (for exemplary learning objectives on the topic of amino acids and proteins, please refer to the Attachment 1, Learning objectives).

The students in the IC intervention groups also received a description of the seminar via Moodle in addition to the named learning objectives. This contained a brief description and not only the content of the biochemistry learning objectives described above but also interdisciplinary, competency-based learning objectives such as communication in a team, in oral examinations and in talks with peers and laymen (see Attachment 1, Seminar description). The time and materials needed were also listed. The content of the biochemistry learning objectives for the IC intervention groups did not differ from that of the control group, the objectives were simply presented briefly once again in the seminar description. In contrast, the competency-based learning objectives were only formulated for the IC intervention groups because only these groups had the necessary free time. 

Accordingly, the IC intervention groups received the materials via the e-learning platform Moodle of the Medical Faculty in Ulm BEFORE the on-site units with exact instructions as to which materials had to be completed by which dates. The traditionally taught control groups received the materials AFTER their on-site units so that they were not disadvantaged with respect to the subsequent biochemistry examination. 

#### 2.4. Data collection

##### 2.4.1. Quantitative data collection

**Evaluation of the teaching concept**

Both the traditional and the IC groups had the opportunity to carry out an evaluation using the official evaluation sheets of the Medical Faculty in Ulm (see Attachment 1, Evaluation sheets). After each course, the students were able to evaluate the course they had attended online for a period of three weeks (evaluation system EvaSys) via the student learning platform Moodle. The questionnaire used for this purpose was conceived based on Rindermann [26]. One question in this evaluation sheet served to differentiate between students in the traditional and IC groups. Furthermore, questions were posed on the organisation, structure and design of the course, learning objectives and content of the course as well as the commitment of the lecturers and the didactic implementation. These questions were rated on a Likert scale from 1 (strongly disagree) to 6 (strongly agree). Students were also able to vote on the need to optimise the course in terms of its organisation/design/structure, methodology/didactics as well as course content (multiple responses were possible). It was also possible to award the course a school grade. 188 (traditional control group) and 32 (IC intervention group) students took part in this voluntary survey. 

Apart from the official evaluation procedure of the Medical Faculty, the IC course was also evaluated by means of a specially conceived questionnaire (see Attachment 1, Evaluation sheets), a paper version of which was issued in the on-site phase II to the IC groups. This questionnaire was drawn up by S.J.K. in close cooperation with M.K. and M.F. It contained two questions in preparation for the on-site phases that could be answered with yes/no. What’s more, the questionnaire included 13 questions that could be evaluated on a Likert scale from 1 (strongly agree) to 5 (strongly disagree). The Likert scale was reversed for the display in the tables shown here. The questions related to:

1. the motivation through the on-site phase O (one questions),
2. the design and the motivation through the preparatory materials (four questions),
3. the coordination of self-learning and on-site phases (one question),
4. the relevance, motivation and increased interest (four questions),
5. the examination and competency training (two questions) and
6. an appraisal of the follow-up time (one question).

A school grade could also be awarded and free text entered a praise and criticism of the course. 

**Evaluation of the teaching films**

One questionnaire was developed for the traditional and one for the IC group to evaluate the teaching films (see Attachment 1, Evaluation sheets). These questionnaires were drawn up by S.J.K. in close cooperation with M.K., M.F. and O.K. The IC groups received these in paper form during the on-site phase II, the traditional groups online in an e-mail sent to the students that contained a link to the online survey via the evaluation platform EvaSys.

**Determination of the learning success**

All of the questions in the official biochemistry exam were evaluated to check the acquisition of knowledge of both the IC and the control group. In a second step, only those eight questions were evaluated whose content correlated with the seminar content. To test the special acquisition of knowledge of the IC group, a formative, written and anonymised test of (case-based) MC (multiple choice) questions (see Attachment 1, Knowledge test) was held at the beginning of the on-site phase O and at the end of the on-site phase II. 

##### 2.4.2. Qualitative data collection

**Evaluation of the IC teaching concept**

In order to optimize the course for coming semesters, the official evaluation sheet of the Medical Faculty in Ulm of the IC group was evaluated qualitatively. The most frequently named issues are shown in the results part. What’s more, both the positive and negative statements were quantified. Comments containing positive and negative statements were counted as positive and negative, in other words, double.

Furthermore, an e-mail survey was carried out after the seminar with participants from the IC group with the question of what motivated the students to prepare for the on-site phase. The statements received were categorised for a qualitative evaluation.

**Evaluation of the teaching films**

The quality of the traditional and IC- groups’ free texts from the questionnaires to assess the teaching films were evaluated. The positive and negative statements were quantified. Comments containing both positive and negative statements were counted as positive and negative, in other words, double.

##### 2.4.3. Data evaluation

The nonparametric Wilcoxon-Mann-Whitney rank sum test (knowledge test, official biochemistry exam) and the t-test for independent random samples (official evaluation sheet) were used as statistical tests. A chi square test (official evaluation sheets) was used for the questions related to the need for optimisation (yes/no questions).

## 3. Results

### 3.1. Comparative analysis of the official evaluation sheet of the Medical Faculty in Ulm

To allow a comparison of the two teaching concepts employed, all students were given the chance to evaluate the seminar electronically after the course with the help of the official evaluation sheet of the Medical Faculty in Ulm. Both groups received identical evaluation questions. 

**Figure 2:**
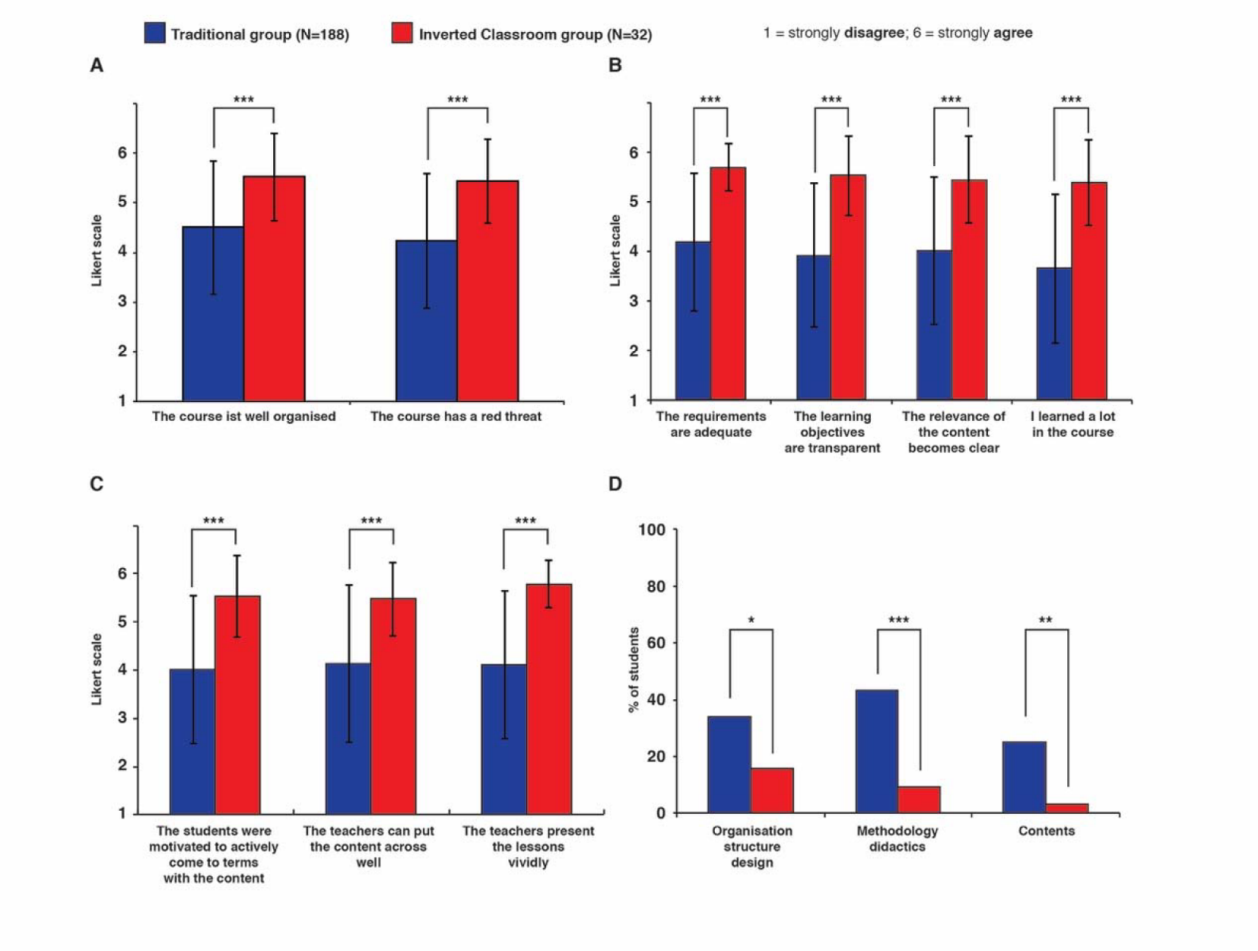
Evaluation results for the traditional versus *Inverted Classroom* group from the official evaluation sheet of the Medical Faculty in Ulm. A-C The items were rated by the students on the basis of a Likert scale from 1 (strongly disagree) to 6 (strongly agree). A. Questions on the organisation, structure and design of the course. ***, p<0.05. B. Questions on the learning objectives and learning content of the course. ***, p<0.05. C. Questions on the commitment of the lecturers and the didactic implementation. ***, p<0.05. D. Vote on the need to optimise the course. Significantly fewer students from the IC group saw a need for optimisation in all fields compared to students from the traditional group. *, p=0.04; **, p=0.01; ***, p=0.001. See the Attachment 1 for individual values.

This evaluation shows that when it came to the question of an overall evaluation of the course, students from the traditional group awarded an average school grade of 2.96 (standard deviation SD=0.80), and those in the IC group an average grade of 1.53 (standard deviation SD=1.29), whereby the difference with a *p*-value of *p*<0.001 is highly significant. The IC method also fared significantly better in all other investigated items with *p*<0.001 (see Figure 2 (Fig. 2); see Attachment 1). Particularly noteworthy here are the determination of the relevance of the learning content (see Figure 2 (Fig. 2), Point B) as well as the motivation of the students to actively come to terms with the learning content (see Figure 2 (Fig. 2), Point C). Furthermore, the IC intervention group rated the more methods-independent items such as the transparency of the learning objectives (see Figure 2, Point B), the mediation of the learning content by the lecturers (see Figure 2 (Fig. 2), Point C) as well as the vivid presentation of the lessons by the lecturers (see Figure 2 (Fig. 2), Point C) significantly better than the traditionally taught control group of students.

When asked about the need for optimisation, the difference between students from the traditional and the IC group became very clear. Special emphasis should be made of the item “Methodology and didactics”. Whereas 43% of students from the traditional group saw a need for improvement here, the figure was only 9% for students from the IC group (see Figure 2 (Fig. 2), Point D).

#### 3.2. Evaluation by the Inverted Classroom group

##### 3.2.1. Quantitative analysis

The evaluations of the IC courses indicate that the students showed a great acceptance of the IC teaching method which were new for them (see Table 1 (Tab. 1)). 

When asked for an overall evaluation of the course, the students awarded a school grade of 1.47 (N=37; standard deviation: 0.64). In addition, 95% of the students said that they had prepared for the on-site phase I and 85% for the on-site phase II. On the whole, the student were satisfied with the preparatory materials; they regarded them as structured, of adequate scope and motivational for the learning process (see Table 1 (Tab. 1)). What’s more, the students from the IC group saw an advantage of the IC method with respect to the follow-up time for the course (see Table 1 (Tab. 1)).

It could also be shown that students considered the course content relevant for their medical studies and increased their interest in biochemistry (see Table 1 (Tab. 1)). The students also recognised that the IC method created free time to train additional communicative competencies such as teamwork and discussions with peers and laymen (see Table 1 (Tab. 1)). Moreover, the students from the IC group felt well-prepared for the pending exams (see Table 1 (Tab. 1)). 

##### 3.2.2. Qualitative analysis 

In order to optimise the course according to the IC method for coming years, a qualitative analysis was also carried out of the free texts of all evaluation sheets from the IC group. The students stated that the IC method was very useful in preparing for the exam, consolidating and repeating the matter, clarifying questions and maintaining a high level of concentration. There follow some excerpts from the free texts:

“The seminar is ideal to prepare for the exam.”

“The teaching videos are very helpful and would also be very useful for other topics or subjects.”

“Because a lot of the matter was repeated as a result of the self-learning process, a lot of it stuck in my mind during the seminar.” 

“Some questions that remained unanswered after the lecture could also be successfully clarified.”

“Thanks to the changing tasks, concentration did not pose a problem over the four hours.”

“A great concept, providing preparatory material so that content could be consolidated during the seminar and a contact person was thus available to clarify any questions!! You have to extend this ....”

However, some students also listed negative points, though these generally related to anchoring the seminar in the timetable as well as the activating units in the on-site phase:

“The time and duration could be improved.”

“I profited least from the presentations of other students.”

“Once would have been enough for the phase in which we had the exam simulation. I found three times a bit too long.”

Summing up, it can be said that with a total of 51 comments, the majority of these, namely 33, were positive. 26 comments, on the other hand, were negative or contained suggestions for further improvements.” 

#### 3.3.Evaluation of the teaching films

##### 3.3.1. Quantitative analysis of the teaching films

It could be shown that all students in the IC intervention group who handed in the questionnaire (N=41) said that they had seen the teaching films before the on-site phase. Similarly, 100% of the students from the IC group (N=41; it should be noted here that one evaluation sheet was not handed in) said that they found the teaching films to be helpful in preparation for the on-site phases. All of the students in the traditionally taught control group (N=72) would have found this helpful too. All of the students who took part in the evaluation would also watch this kind of teaching film for other courses (N=113). The students awarded the three teaching films an average school grade of 1.38 (N=108).

All of the students thought that the learning content in the teaching films was well structured, understandable and on an appropriate level (see Table 2 (Tab. 2)). 

##### 3.3.2. Qualitative analysis of the teaching films

Since the existing teaching films are to be optimised in future and further biochemistry teaching films developed, the quality of the free texts in the evaluation sheets on the teaching films were also evaluated. The students considered the visualisation of complex relationships and the many different ways in which the films could be used, for example as an introduction to or refresher element for a certain subject matter, to be very helpful. There now follow some positive statements about the teaching films by way of example:

“Very good teaching films that bring across the content in a pleasant way.” 

“... The visualisation of complex process in a simple, understandable way is excellent.”

“Very well structured and an appropriate amount of matter.”

“The explanations are perfect because they present the essentials concisely, and the animations make it easier for you to visualise these. Just great!!!”

“They are suitable to both acquire new knowledge and to refresh what has already been learned.”

A total of 50 students left only positive comments on the teaching videos.

The most commonly named negative aspect was the speech tempo. Some students would have liked a faster speech tempo. A total of 20 students made suggestions on how to optimise the teaching videos, whereby the majority of students still found the basic idea very good. 

#### 3.4. Acceptance of the IC method: results of a qualitative survey

On account of the large number of students in the IC group who came to the on-site phase prepared, a qualitative e-mail survey was carried out after the seminar among these 42 participants in the IC intervention group to find out what had motivated the students to prepare for the on-site phase. A total of ten students took part in the voluntary survey, whereby only the most commonly named points will be listed here:

- the preparation through the good quality and short teaching films as well as self-learning control tasks (n=8)
- the preliminary meeting (on-site phase 0) in which the necessity of preparation, the “why” behind the new conception as well as the organisation and sequence of the seminar were explained (n=7)
- the high motivation of the lecturers (n=5)
- the covering of matter relevant for the exam (n=3)

#### 3.5. Measurement of the learning success

##### 3.5.1. Results of the knowledge test in the IC group

**Figure 3:**
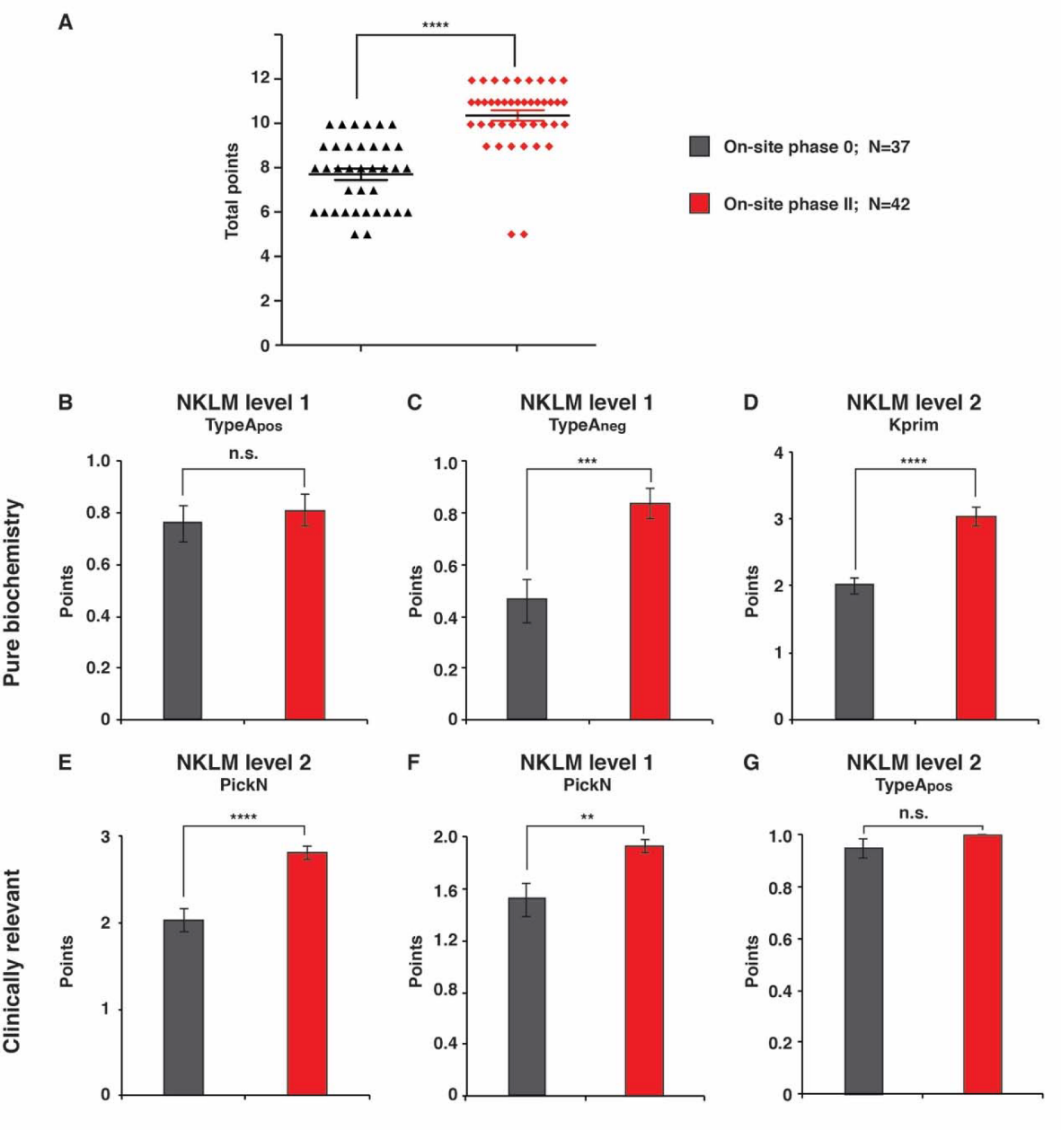
Results of the knowledge test A. Results of the knowledge test held with the IC groups in the on-site phases 0 and II. The maximum number of points that could be achieved was 12. 37 students took part in the knowledge test in on-site phase 0 and 42 in on-site phase II. B-G. Classification of the questions from the knowledge test as purely biochemistry questions (B-D) and questions with a clinical relevance (E-G). Competency level 1 (factual knowledge) and 2 (reasoning knowledge and know-how) according to the NKLM as well as the types of questions are also shown. The Y-axis shows the maximum possible number of points for each questions. p-value, ** p=0.01; *** p=0.001; **** p=0.0001; n.s., not significant.

In order to determine whether there had been an increase in not only the motivation and satisfaction of the students in the IC intervention group but also in the learning success between the beginning and end of the course, a knowledge test was carried out with the IC group at both the beginning, i.e. in on-site phase 0, and thus before the self-learning phase I, and the end of the course. This test asked not only normal MC questions of the type A_pos_ and A_neg_ but also MC questions of the type K_prim_ and PickN along with case-based questions with a clinical relevance; this called for not only a rethink of the otherwise familiar MC questions but also an application of biochemistry principles for clinical issues. In addition, competency levels 1 (factual knowledge) and 2 (reasoning knowledge or know-how) from the NKLM were tested. This test showed that the students in the IC group experienced a significant acquisition of knowledge equally on both competency levels (see Figure 3 (Fig. 3)). It is also striking that the students already coped well with the type A_pos_ MC questions at the time 0, i.e. answered the majority of these correctly. In contrast, a significant acquisition of knowledge was recorded, particularly with the other types of question.

##### 3.5.2. Results of the official biochemistry exam

The next step investigated whether a difference in the learning success could be seen between the students from the IC intervention group and those in the traditional control group. To this end, the results of the official biochemistry exam, held two weeks after the integrated seminar, were evaluated. This written biochemistry consisted of 20 multiple choice (MC) questions of the type A_pos_ and A_neg_, which mainly tested knowledge on NKLM competency level 1. 

The results of all questions were initially evaluated. It could hereby be seen that the students from the traditional control and the IC groups achieved an average of 13.1 from 20 possible points (control group: n=299, standard deviation: 2.8; IC group: n=40, standard deviation: 3.0), so that there was no difference in the acquisition of knowledge. 

Eight of these 20 questions were thematically based on the content of the integrated seminar and could be answered upon a participation in the integrated seminar. The results of only these eight questions were then analysed to measure the learning success from the integrated seminar. This analysis showed that the students in the IC group achieved an average of 5.07 from a possible 8 points (n=40; standard deviation: 1.49), the students in the traditional group an average of 5.05 points (n=299; standard deviation: 1.55), which does not constitute a significant difference.

Accordingly, the students in both groups had an equally good learning success relative to answering MC questions in the subject biochemistry.

## 4. Discussion

The data from our study shows that

1. the motivation and satisfaction of second semester students of human medicine can be greatly increased through the use of the e-learning-based IC method in the biochemistry seminar,
2. the IC method is accepted by students of human medicine in the second pre-clinical semester,
3. there is no significant difference between students from the IC and the traditional group when it comes to the learning success for biochemistry content,
4. with a good organisation, good preparatory materials and motivated lecturers, the IC method can be implemented very well in a biochemistry seminar in the pre-clinical stage of a human medicine degree,
5. the added value of the IC method consists of teaching additional, competency-based learning objectives.

### 4.1. Motivation and satisfaction with the IC method

Our data shows that motivation and satisfaction are greatly increased by the IC method. This correlates with the findings of other studies from both the pre-clinical and clinical stage of studies [3], [9], [21]. We were also able to show that all of the students who took part would welcome further teaching films in other courses too, indicating a high level of satisfaction with teaching films provided. Kiviniemi and colleagues also reported a similar effect; in their study, 83% of medical students preferred a “blended learning” approach over purely face-to-face teaching [14]. In a next step, it would be interesting to expand the IC method to all of the participants in the seminar as well as to other courses and then determine the motivation and satisfaction.

#### 4.2. The acceptance of the IC method in the biochemistry seminar within the pre-clinical stage of studies

This study has clearly shown that second semester students of human medicine display a great acceptance of the IC method in a biochemistry seminar.

A big obstacle for a successful implementation of the IC method is to motivate students to prepare for the on-site phase in the self-learning phase. One way to achieve this would be to introduce an admission test that has to be passed to participate in the on-site phase. An admission test was deliberately not included when planning the course because we believe that this would take us further away from our goal of motivating students and capturing their imagination for biochemistry. This study relied on an information event (on-site phase 0) before the actual seminar itself that explained the background to the change to the course based on the evaluation results of the previous years. Apart from a presentation of the teaching films, the students were also told that the on-site phases could only be used effectively if they prepared for these. To our surprise, the analyses of the evaluation sheets showed that 85-95% of the students had prepared for the on-site phase, indicating a successful implementation of the IC method. 

#### 4.3. The learning success through the IC method

The results of the official biochemistry exam, held two weeks after the seminar, were compared on the basis of all of the questions and on the basis of eight questions relevant for the seminar to compare and measure the learning success of the students in both groups. We were unable to find a significantly better result for students from the IC group compared to those from the control group. This is in line with the results of other teams who were also unable to show an increased acquisition of knowledge using the IC method compared to traditional teaching methods [12], [21]. Nor is this surprising in our study design since firstly, the time taken up by the seminar compared to the overall teaching and learning time for biochemistry within two semesters is fairly low, and secondly, the control group were also shown the teaching films after their seminar, but before the exam. This was important to give all of the students an equal chance to do well in the official biochemistry exam. The past years have also shown that students of human medicine in Ulm in their pre-clinical stage of studies are very well prepared for the M1 state examination because the IMPP results are above the national average. For example, in 2014 only 4.7% (German national average: 7.8), and in 2015 only 3.3% of students (German national average: 8.1) failed the written part of the M1 state examination. In the fields of chemistry/biochemistry/molecular biology, the average overall score in 2014 was 74.7 points (German national average: 72.3), and in 2015 this was 78.7 points (German national average: 75). This data shows that the students are already very well prepared for the MC exams by the traditional teaching method in the seminar too. It can therefore be concluded that the level of biochemistry teaching at Ulm University is already so high that the IC method would not contribute directly to an increase in the acquisition of knowledge with respect to answering MC questions in this small course. It is planned to extend the IC method to other biochemistry courses so that its influence on the learning can be investigated on a larger scale.

As a second approach we carried out a knowledge test with the IC intervention group, once before and once at the end of the seminar. A significant acquisition of knowledge on both NKLM competency levels 1 and 2 could be recorded. 

The sum total of both tests (official biochemistry exam, knowledge test in the IC group) was that the students from the IC intervention group did not fare either better or worse in an MC exam than the traditional group.

The question arose at this point as to whether an MC exam was a suitable test at all to measure the learning success in this context. The IC course we implemented was more concerned with teaching students in the on-site phases additional competencies such as communication in a team, in oral exams and in talks with peers and laymen, and not with the acquisition of further content-related topics or preparation for MC questions. Hence, it would be very interesting in future to determine whether students from the IC group did better in these competencies than students from the traditional group. In order to measure the competency “communication in oral exams”, for example, standardised oral exams could be held and the results of students from the IC group then compared with those of students from the traditional group. An OSCE ward with a standardised patient [4] would be conceivable to test the competency “communication with a layman”. It should also be pointed out here that solving MC questions calls for at least conceptual knowledge. Since the IC method focuses on the application of factual knowledge, it would be important in future to compare the learning success of students taught by the traditional versus the IC teaching method by testing their conditional knowledge (reasoning knowledge). This could take the form of case-based or problem-solving questions [27], [28].

#### 4.4. Teaching films in biochemistry

We stuck to the guidelines of Guo et al. [8] when preparing the teaching films. Thus, we made sure that none of the films lasted longer than 10 minutes, complex biochemical processes were represented by simple illustrations and that only the most important terminology appeared in writing. Contrary to Guo et al.’s recommendation, the lecturer was not superimposed so as not to distract the students’ attention from the illustrations and terminology. We chose a lecturer already known to the students from the main lectures as a speaker so that they were sure to be able to identify them. We decided on a speech tempo similar to that of the lecture tempo. As the evaluations showed, the teaching films scored very well. Whereas some students praised the tempo, the majority were in favour of a faster tempo. This is in line with the results obtained by Guo et al., who discovered that a faster speech tempo encourages a higher commitment from the viewers [8]. A faster tempo is therefore recommended, whereby we see no problem for the students who want to take notes because the films can be viewed repeatedly. Another point to be considered is whether the slides from the films should be provided so that students can take notes and reproduce drawings faster. 

Apart from the specially created teaching videos, we also provided the students with documentary films on the three clinical symptoms for preparation. The students said that they did not remember a lot about these because they did not consider them to be relevant. Consequently, we recommend the preparation of own teaching videos in future that also match the content of the on-site phase 100%.

#### 4.5. Benefits of the IC method for competency-based training in medical studies

One big advantage of the IC method is that exporting factual knowledge in the self-learning phase creates free time in the on-site phase that can be put to other use. In this study, this free time was used to teach competency-based learning objectives. Thus, apart from the biochemistry learning objectives, communication competencies in a team, in oral exams and in talks with peers or laymen (medical communication) were strengthened (see Attachment 1, Seminar description). The evaluations confirmed the perception of these intended learning objectives amongst the students in the IC intervention group. In contrast, these competency-based learning objectives were not, or at least not the main goal of the traditional course, so that the phases of student attendance were used/had to be used for the acquisition of facts.

The very good grade awarded to the overall course by the IC intervention group proves that the students were very satisfied with the IC method including the content taught. With respect to the recently published *Masterplan Medizin 2020* [https://www.bmbf.de/de/masterplan-medizinstudium-2020-4024.html], the IC method offers a big opportunity to integrate the strengthening of competency-based training this advocates, such as social and communicative competencies, in teaching practice.

#### 4.6. Limitations of the study

The present study was limited by certain factors. One limitation is the inhomogeneous team of lecturers. The traditional student group was taught by a team of mainly experienced lecturers who had held the seminar in this form for several years (12 out of the 14 control groups). This team was complemented by an inexperienced lecturer with no previous experience in either the field of teaching or this specific seminar (2 out of 14 control groups). The IC groups (2 groups) were taught by a lecturer with a lot of teaching experience. However, because this lecturer held this seminar including the three clinical symptoms for the first time, she was at a disadvantage compared to the team of largely experienced lecturers for the traditional groups with respect to the clinical context. Furthermore, the lecturer for the IC intervention group displayed a high level of motivation, which could have been transmitted to the students irrespective of the teaching method. However, it should also be noted that the majority of lecturers in the team for the traditional control group were also motivated. Nevertheless, we were well aware in advance that the successful employment of a new teaching method depends on the lecturer [10]. This was also confirmed by the students in an e-mail survey, where some of the students said that the high motivation of the lecturer was one reason for their preparation for the on-site phases. All in all, however, an inhomogeneous team of lecturers reflects the teaching reality without any artificial scenario.

One further limitation of the study were the rooms. Because two groups were always taught in parallel, they also taught in different rooms. Thus, the seminar for the two IC groups was held in a room with daylight. Of the 14 control groups, eight were taught in an interior room without daylight and six in the same room as the IC groups. Otherwise the rooms’ layout and equipment were similar. 

Another limitation of the study was the use of the knowledge test for only the IC group. This was due to personnel restrictions. The knowledge test served to measure the acquisition of knowledge of students from the IC intervention group in terms of the NKLM competency levels 1 and 2 to rule out the possibility of no increase in biochemistry knowledge on the part of the students due to a lack of preparation in the self-learning phase. The positive increase in knowledge comes as no surprise in view of the participation of all of the students in the self-learning phase.

The comparison of the acquisition of knowledge (IC versus control group) was carried out in this study by analysing specific questions in the official biochemistry exam. Special attention was paid here to the appropriation of biochemistry knowledge on NKLM competency level 1. As has already been mentioned above, a knowledge test at the beginning and the end of the course should be carried out with all student participants in a future study.

## 5. Conclusion and outlook

The study performed here shows that the Inverted Classroom method is very well accepted amongst pre-clinical students in the biochemistry seminar. One parameter that turned out to be critical in the course of a qualitative evaluation is that the quality of the materials provided must be high. It also appears to be important that the concept be explained in detail to the students. During a quantitative evaluation, the Inverted Classroom method fared much better that the traditional teaching method with respect to all of the parameters recorded. We were unable to identify an increased acquisition of knowledge by the tests used in this study. It would certainly make sense to use better instruments to measure the success, such as a standardised oral exam or problem-solving tasks, for this purpose in future so as to record the additional communicative competencies that have been learned or the conditional knowledge that has been appropriated. Summing up, it can be concluded that the IC method is a suitable strategy to implement the competence orientation of medical studies in the pre-clinical phase as published in the Masterplan Medizin 2020 [https://www.bmbf.de/de/masterplan-medizinstudium-2020-4024.html].

## 6. Financial support

The project was supported by the Medical Faculty of Ulm University with funds from the “Sonderlinie Medizin” of the State of Baden-Württemberg, Germany.

## Competing interests

The authors declare that they have no competing interests.

## Supplementary Material

Details of the teaching concept and the teaching content – in German

## Figures and Tables

**Table 1:**
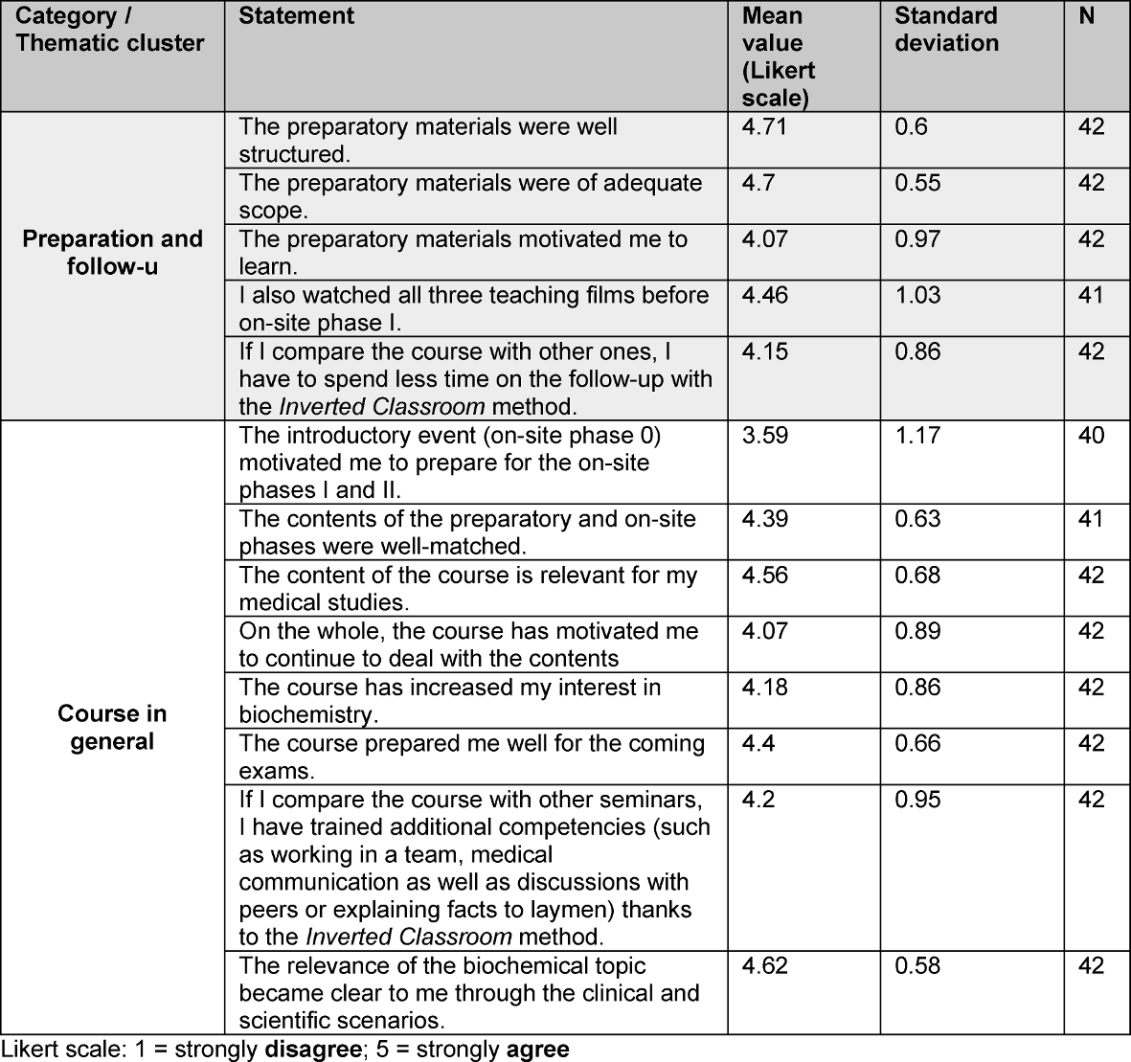
Summary of results from the evaluation of the overall course Integrated Seminar Module 6 – Pathobiochemistry II: ”From a gene to protein” of the *Inverted Classroom* group

**Table 2:**
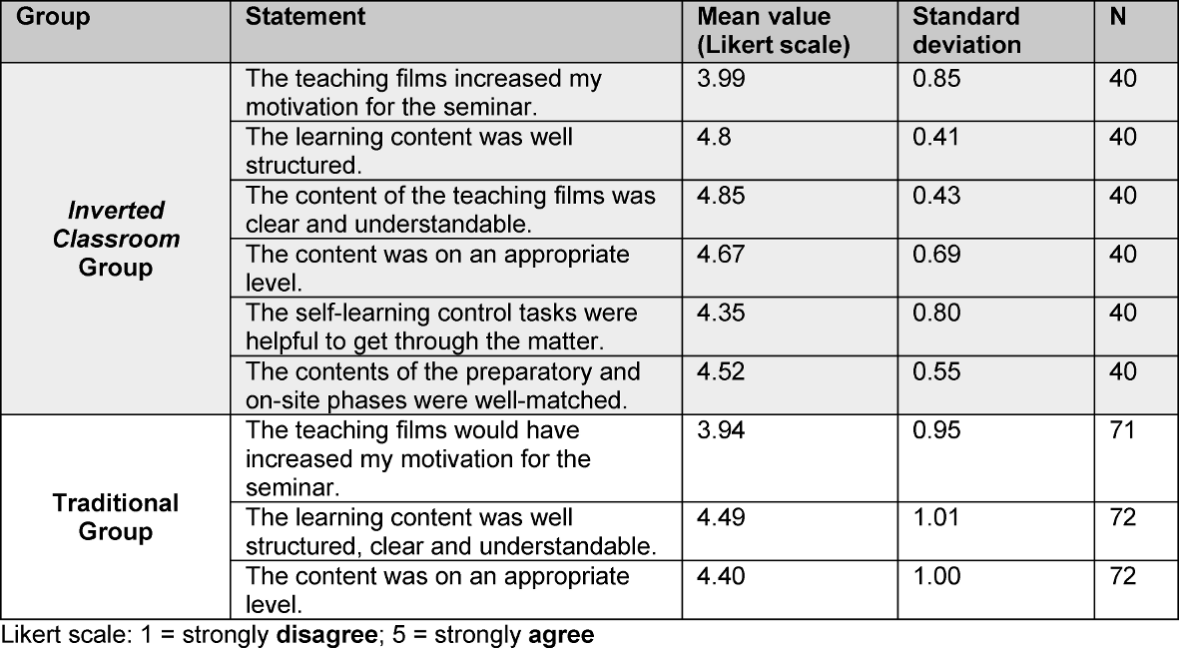
Results from the evaluations of the pilot teaching films
