# Supplementary material for: Concept and benefits of the Inverted Classroom method for a competency-based biochemistry course in the pre-clinical stage of a human medicine course of studies
Source: GMS J Med Educ. 2017 Aug 15;34(3):Doc31. doi: 10.3205/zma001108 (PMC5569983; doi:10.3205/zma001108)
Supplement: Details of the teaching concept and the teaching content – in German [file JME-34-31-s-001.pdf]

Anhang

Konzeption und Nutzen der *Inverted Classroom*-Methode für eine kompetenzorientierte Biochemie Lehrveranstaltung im vorklinischen Studienabschnitt der Humanmedizin

Susanne J. Kühl[1,*], Matthias Toberer[1], Oliver Keis[2], Daniel Tolks[3], Martin Fischer[3] und Michael Kühl[1]

1 Institut für Biochemie und Molekulare Biologie, Universität Ulm, 89081 Ulm
2 Studiendekanat Medizinische Fakultät Ulm, Universität Ulm, 89081 Ulm
3 Institut für Didaktik und Ausbildungsforschung in der Medizin, Ludwig-Maximilians-Universität München, 80336 München

*** Korrespondierende Autorin:**

Dr. Susanne J. Kühl
Institut für Biochemie und Molekularbiologie
Universität Ulm
Albert-Einstein-Allee 11
89081 Ulm, Deutschland
susanne.kuehl@uni-ulm.de

Zusätzliche Tabelle: Evaluationsergebnisse im Vergleich der traditionellen versus der *Inverted Classroom* Gruppe aus dem offiziellen Evaluationsbogen der Medizinischen Fakultät Ulm.

**Aussage**	***Inverted Classroom* Gruppe**			**Traditionelle Gruppe**			**p-Werte** (Vergleich IC- mit traditionellen Gruppe)
	Mittelwert (Likert-Skala)	N	Standardabweichung	Mittelwert (Likert-Skala)	N	Standardabweichung	
Die Lehrveranstaltung ist gut organisiert.	5,53	32	0,88	4,50	187	1,35	<0,001
Die Veranstaltung hat eine gute Gesamtstruktur ("Roter Faden").	5,44	32	0,84	4,24	186	1,36	<0,001
Die gestellten Anforderungen sind adäquat.	5,69	32	0,47	4,18	186	1,38	<0,001
Die Lernziele der Lehrveranstaltung sind transparent.	5,53	32	0,80	3,91	186	1,45	<0,001
Die Relevanz der Lerninhalte wird deutlich.	5,44	32	0,88	4,01	186	1,49	<0,001
Ich habe in der Lehrveranstaltung viel gelernt.	5,38	32	0,87	3,65	186	1,50	<0,001
Die Studierenden werden motiviert, sich aktiv mit den Lerninhalten auseinanderzusetzen.	5,53	32	0,84	4,01	187	1,54	<0,001
Die beteiligten Lehrenden können die Lehrveranstaltungsinhalte gut vermitteln.	5,47	32	0,76	4,14	186	1,63	<0,001
Die beteiligten Lehrenden gestalten den Unterricht anschaulich (mit praktischen Bezügen, Beispielen, etc.).	5,78	32	0,49	4,11	185	1,54	<0,001

1 Exemplarische Lernziele zum Thema Aminosäuren und Proteine

Lernziele Hauptvorlesung Biochemie 1. Semester

Thema: Aminosäuren und Proteine

Dozent: Prof. Dr. Michael Kühl

Theoretische Inhalte in blau

Methodische Inhalte in olive

Medizinische Relevanz/Krankheitsbilder in rot

Kompetenzstufen in schwarz (Details siehe Datei „Definition der Kompetenzstufen“)

Nach Besuch der Vorlesungsreihe sollten die Studierenden in der Lage sein, ...

1. **die Klassifizierung, die Struktur und die Eigenschaften von AS zu erläutern.**
   Theoretische Inhalte: D/L-Nomenklatur, Buchstabencodes für AS, proteinogene und nicht-proteinogene AS, Pufferwirkung von AS, Titrationskurven, Isoelektrischer Punkt.
   Kompetenzstufe: 2

2. **den isoelektrischen Punkt einer Aminosäure mit potentiell geladenen Seitenketten zu berechnen.**
   Kompetenzstufe: 2

3. **die Peptidbindung zu erläutern.**
   Theoretische Inhalte: Bildung, Doppelbindungscharakter, cis/trans-Konfiguration von Peptidyl-Prolyl-Bindungen.
   Kompetenzstufe: 2

4. **den Aufbau, die Funktion und die Eigenschaften von Peptiden und Proteinen zu erläutern.**
   Theoretische Inhalte: Primärstruktur, Sekundärstrukturen mit α-Helix, β-Faltblatt, β-Turn und den stabilisierenden Bindungen, Tertiärstruktur mit den stabilisierenden Bindungen, Proteinmotiv, Proteindomäne, Quartärstruktur: Untereinheiten.
   Kompetenzstufe: 2

5. **den Prozess der Proteinfaltung zu erläutern. Die Studierenden sollten Erkrankungen die Proteinfaltung betreffend nennen und deren biochemischen Hintergrund auf die jeweiligen Erkrankungen anwenden können.**

   Theoretische Inhalte: Hierarchische Faltung von Proteine; Aufbau, Funktion und Eigenschaften von Chaperonen.

   Krankheitsbilder: spongiforme Enzephalopathien (Prionenerkrankung), Amyloidosen

   Kompetenzstufe: 3

6. **die Begriffe Denaturierung und Renaturierung zu erklären.**

   Theoretische Inhalte: Aufbau, Funktion und Eigenschaften des Proteasoms

   Kompetenzstufe: 2

7. **den Prozess und die Funktion der Polyubiquitinierung zu erklären.**

   Kompetenzstufe: 2

8. **den Aufbau, die Funktion und die Eigenschaften eines Liganden zu erläutern.**

   Theoretische Inhalte: prosthetische Gruppe

   Kompetenzstufe: 2

9. **die Interaktion zwischen Ligand und Rezeptor zu erklären.**

   Theoretische Inhalte: AS Interaktionen

   Kompetenzstufe: 2

10. **die Struktur und Funktion von Myoglobin und Hämoglobin zu erläutern. Die Studierenden sollten Erkrankungen Myoglobin und Hämoglobin betreffend nennen und deren biochemischen Hintergrund auf die jeweiligen Erkrankungen anwenden können.**

    Theoretische Inhalte: Konformation, Kooperativität, Allosterie, Hill-Koeffizient, Bohr-Effekt, Unterschied zwischen embryonalem und fetalem Hämoglobin

    Krankheitsbilder: CO-Vergiftung, Sichelzellenanämie, Thalassämie

    Kompetenzstufe: 3

11. **den Aufbau, die Funktion und die Eigenschaften von α-Keratin zu erläutern.**

    Kompetenzstufe: 2

12. **den Aufbau, die Funktion und die Eigenschaften von Kollagen zu erklären. Die Studierenden sollten Erkrankungen Kollagen betreffend nennen und deren biochemischen Hintergrund auf die jeweiligen Erkrankungen anwenden können.**

Theoretische Inhalte: Tripelhelix

Krankheitsbilder: Skorbut, Ehlers-Danlos-Syndrom, *Osteogenesis imperfecta.*

Kompetenzstufe: 3

13. **für bestimmte Fragestellungen zur Analyse von AS und Proteinen geeignete methodische Verfahren zu nennen und zu erklären.**

    Methodische Inhalte: Nachweis von AS mit Ninhydrin, Proteinsequenzierung, SDS-PAGE, Western Blot, ELISA, Röntgenstrukturanalyse, subzelluläre Lokalisation: Antikörper, GFP

    Kompetenzstufe: 2

2 Seminarbeschreibung

Integriertes Seminar Modul 6 - Pathobiochemie II: „Vom Gen zum Protein“

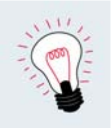

Kurzbeschreibung

Das Seminar gibt einen Überblick über die Proteinbiosynthese. Störungen dieses Prozesses resultieren oftmals in schweren Erkrankungen. Im Seminar beziehen wir uns bespielhaft auf die Synthese des Proteins Kollagen Typ I, wobei Störungen in der Kollagensynthese Krankheitsbilder wie *Osteogenesis imperfecta,* Ehlers-Danlos-Syndrom und Skorbut hervorrufen können. Neben der Vermittlung biochemischer Grundlagen sollen zudem Schlüsselkompetenzen wie das Arbeiten in einem Team, die Kommunikation während einer mündlichen Prüfung sowie Gespräche mit Fachkollegen und Patienten gestärkt werden.

Dozentin und Lehrbeauftragte

Dr. Susanne Kühl (susanne.kuehl@uni-ulm.de)

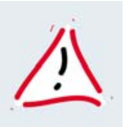

Lernziele

Inhaltliche Lernziele

Nach der Veranstaltung sollen die Studierenden in der Lage sein,

1. die Proteinbiogenese im Allgemeinen und von Kollagen Typ I im Speziellen zu erläutern.
2. den Aufbau und die Eigenschaften von Kollagen Typ I zu visualisieren.
3. das Krankheitsbild *Osteogenesis imperfecta* (OI) hinsichtlich Klassifizierung, Symptomatik und Schweregrad darzulegen.
4. Vor- und Nacheile diagnostischer und therapeutischer Ansätze zu OI zu erklären.
5. das Ehlers-Danlos-Syndrom und Skorbut hinsichtlich Symptomatik und biochemischen Ursachen zu erläutern.
6. biochemische Hintergründe der drei Krankheitsbilder sowohl vor Experten als auch für Laien verständlich zu beschreiben.

Kompetenz-orientierte Lernziele

Während der Veranstaltung wird die Kommunikationsfähigkeit der Studierenden in

1. einem Team (*durch Kleingruppenarbeiten*)
2. mündlichen Prüfungen (*durch Simulation und Feedback*)
3. Diskussionen mit Fachkollegen (*durch Simulation und Feedback*)
4. Gesprächen mit Patienten / Fachfremden (*durch Simulation / Feedback*)

trainiert.

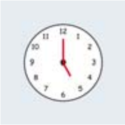

Zeitaufwand

Selbstlernphasen: 2x ca. 45 Minuten

Präsenzphasen: 2x 4 Stunden

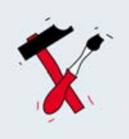

Mitzubringendes Material für die Präsenzphasen (Ausdruck oder elektronisch)**:**

- Seminarbeschreibung
- Schreibutensilien
- Biochemie-Lehrbuch
- Arbeitsblätter Vorbereitungsgruppen 1-3
- wenn gewünscht: Präsentationsfolien (werden am Abend vorher auf Moodle online gestellt)

Notizen für die Evaluation Tag 1:

Notizen für die Evaluation Tag 2:

3 Selbstlernaufgaben

Aufgaben zu Film 1

Aufgabe 1: Bitte beschriften Sie die Abbildung zur Transkription.

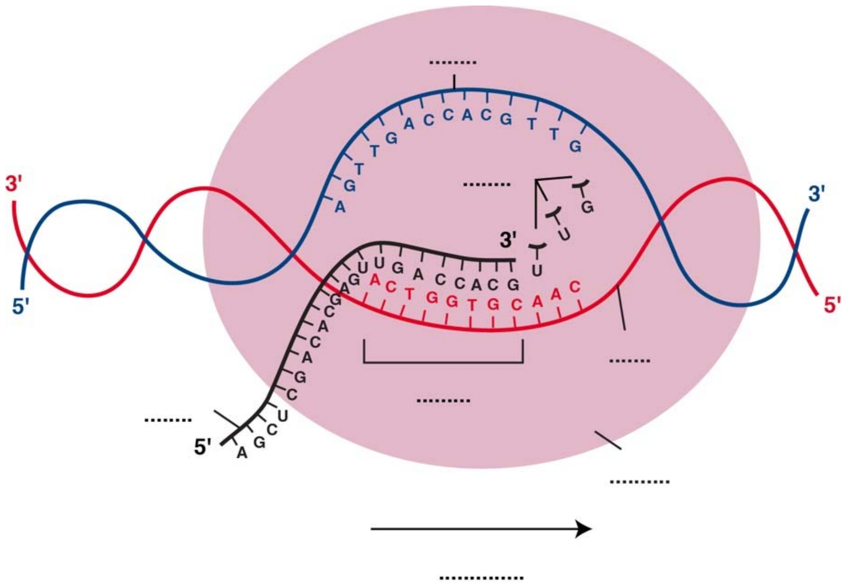

Aufgabe 2: Bitte beschriften Sie die Abbildung zur Translationsinitiation.

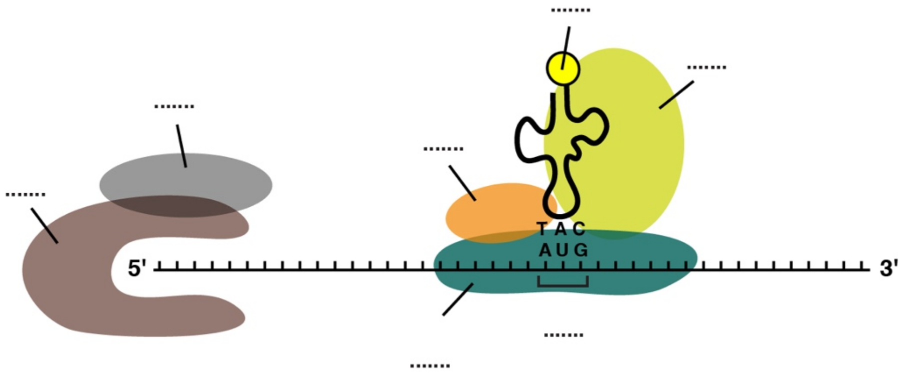

Aufgabe 3: Bitte markieren Sie die (<u>eine</u>) korrekte Aussage in der rechten Spalte mit einem „X“.

	Bei der Regulation der Transkription.....	
**A**	können die Transkriptionsfaktoren sowohl hemmend als auch aktivierend wirken.	
**B**	spielen die Faktoren eIF2 und eIF4 eine wichtige Rolle.	
**C**	ist das Startkodon ATG beteiligt.	
**D**	wirken die cis-regulatorischen Enhancer Elemente hemmend.	
**E**	hat die RNA-Polymerase keine Funktion.	

Aufgaben zu Film 2

Aufgabe 1: *Bitte beschriften Sie die Abbildung zur Proteinbiosynthese am rER.*

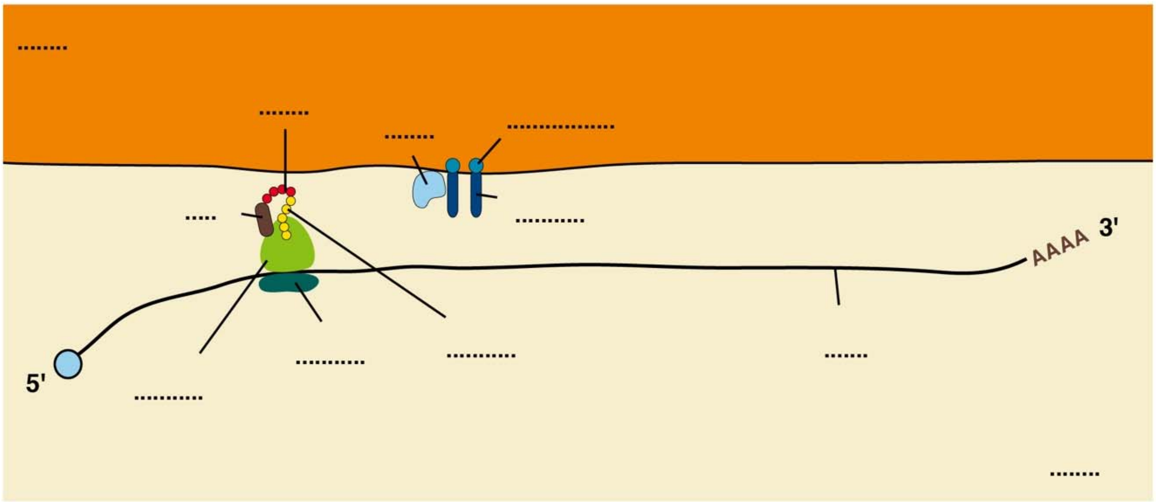

Aufgabe 2: *Bitte beschriften Sie die Abbildung zum Vesikeltransport.*

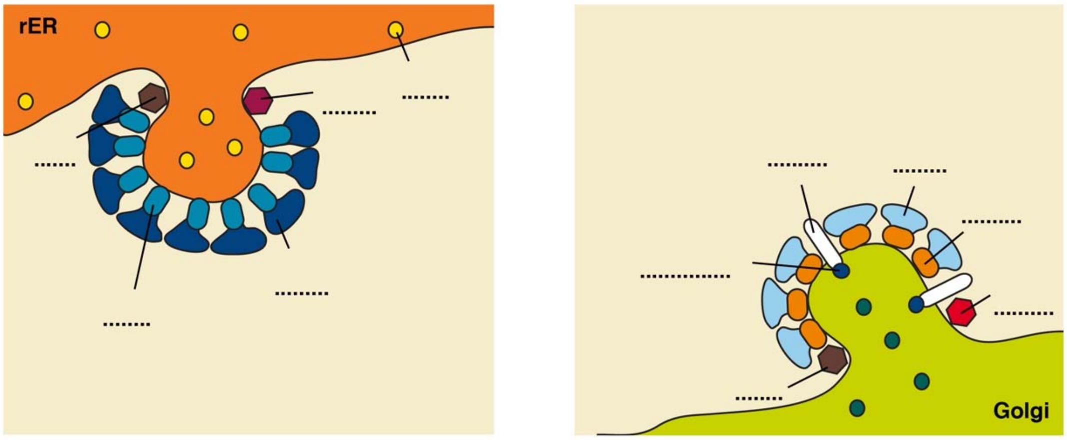

Aufgabe 3: *Bitte lösen Sie folgende MC Frage.*

*Bitte markieren Sie die (<u>eine</u>) Aussage, die **<u>NICHT</u>** korrekt ist, in der rechten Spalte mit einem „X“.*

**A**	SRP steht für *signal recognition particle*.	
**B**	Chaperone unterstützen die Proteinfaltung im rER.	
**C**	Die Proteindisulfid-Isomerase sorgt für die korrekte Bildung von Disulfidbrücken im Protein.	
**D**	Proteine werden an Asparaginreste O-glykosidisch mit Kohlenhydrateinheiten modifiziert.	
**E**	Der Golgi-Apparat wird in cis, medianer und trans Golgi unterteilt.	

Aufgaben zu Film 3

Aufgabe 1: *Bitte beschriften Sie die Abbildung zur Hydroxylierung.*

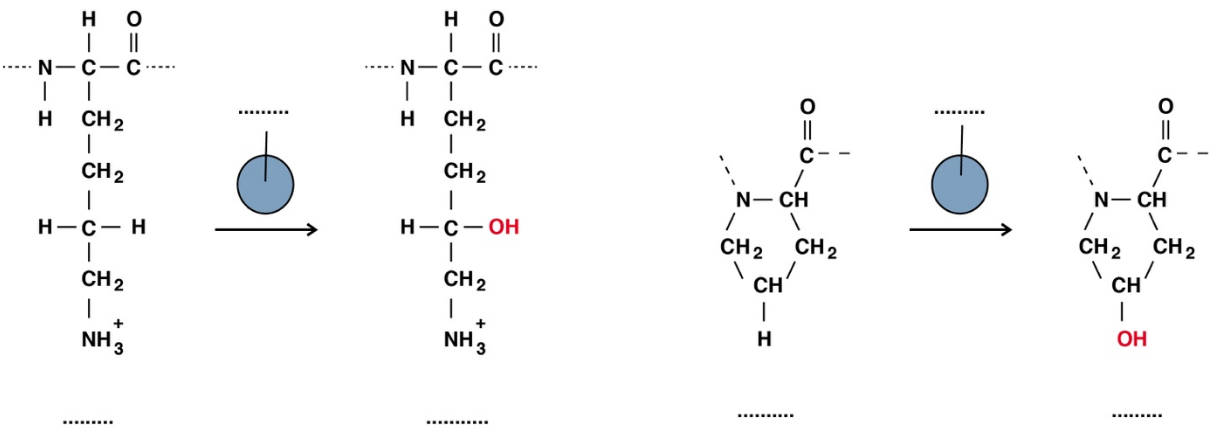

Aufgabe 2: *Bitte beschriften Sie die Abbildung zum Kollagen.*

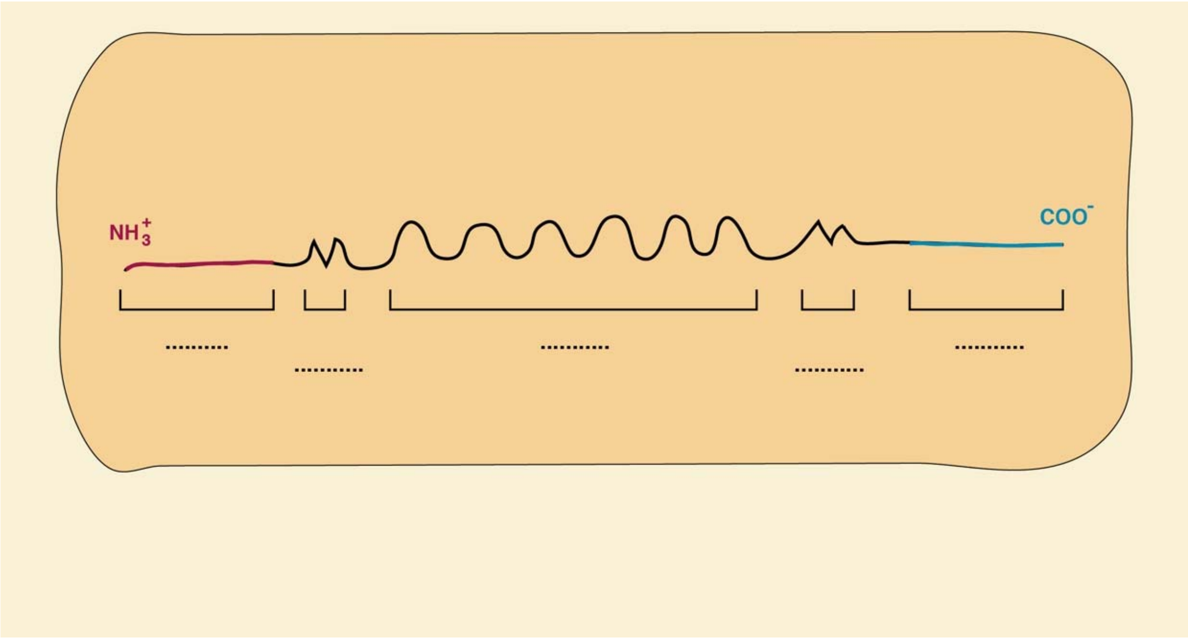

Aufgabe 3: Bitte entscheiden Sie für ***<u>JEDE</u>*** *Aussage, ob sie korrekt ist oder nicht. Markieren Sie dies entsprechend in der rechten Spalte mit einem „X“.*

Bei der Kollagensynthese.....	korrekt	nicht korrekt
werden Kohlenhydrate über eine N-glykosidische Bindung an einen Hydroxylysylrest angehängt.		
spielen für die Tripelhelixbildung Disulfidbrücken am N-Propeptid eine entscheidende Rolle.		
katalysieren Peptidasen eine Abspaltung der Propeptide, wodurch Tropokollagen entsteht.		
katalysiert die Lysyloxidase die Umwandlung von Hydroxylysin zu Hydroxyallysin.		

4 Abläufe der Präsenzphasen

Zusätzliche Tab. 2: Ablauf Präsenzphase I. Dargestellt sind die Zeiten inkl. der Dauer der einzelnen Schritte, die Themen, didaktische Methoden, Ziel der jeweiligen didaktischen Methoden, Kompetenzen sowie Kompetenz- und Arztrollen nach NKLM und die Kompetenzebenen nach Bloom, 1984.

Zeit	Thema	Didaktische Methode	Ziel der didaktischen Methode	ICAP	Kompetenz / Medizinisches Wissen	Kompetenz- / Arztrollen	Kompetenzebenen (Bloom, 1984)
14:00-14:10	Begrüßung und Vorschau	Agenda / Lernziele (Flipcharts an der Wand)	Übersicht des Tagesablauf				
14:10-14:35	*Osteogenesis imperfecta* Ursachen Symtome / Diagnose / Therapie (Was fällt Ihnen dazu ein?)	Gemeinsame Erarbeitung am Flipchart	Aktivierung von Vorwissen aus Selbstlernphase I	aktiv	Prinzipien der Pathogenese und Pathomechanismen	Medizinischer Experte	Wiedergeben
14:35-15:00	*Osteogenesis imperfecta* (Biochem. Hintergrund, Klassifizierung und Symptome)	Impulsvortrag (Power Point)	Informationsvermittlung mit Aktivierung	passiv	Prinzipien der Pathogenese und Pathomechanismen	Medizinischer Experte	Verstehen
15:10-15:15	Erläuterung und Ausgabe der Gruppenarbeiten	Prüfungsrelevante Themen mit klarer Aufgabenstellung	Gruppen im Vorfeld durch Dozentin eingeteilt	passiv			
15:15-15:55	Gruppe 1: Transkription<br>Gruppe 2: Translation<br>Gruppe 3: Synthese ins ER<br>Gruppe 4: Vesikeltransport<br>Gruppe 5: Synthese Kollagen-Tripelhelix Teil 1<br>Gruppe 6: Synthese Kollagen-Tripelhelix Teil 2	Gruppenarbeit<br><br>(Aufgabenstellung ähnlich einer mündlichen Prüfung: grafische Darstellung auf Flipchart)	Aktivieren und Vertiefen von biochemischem Grundlagenvorwissen aus Selbstlernphase I und Vorlesung 1. Semester	konstruktiv	Prinzipien normaler Struktur und Funktion	Medizinischer Experte<br>Mitglied im Team	Anwenden
15:55-16:10	Pause						
16:10-17:15	Gruppenthemen siehe oben	Simulation einer Prüfung (6x) mit Feedbackbogen	Anwenden der biochemischen Grundlagen	transaktiv	Prinzipien normaler Struktur und Funktion	Medizinischer Experte<br>Kommunikator	Anwenden
17:15-17:45	Ursache, Schweregrad und Diagnostik von OI (anhand eines klinischen Falls)	Interaktiver Vortrag (Power Point mit integrierten Aufgaben und Diskussion)	Informationsvermittlung mit Aktivierung<br>Anwenden, vertiefen der biochemischen Grundlagen	passiv, aktiv, konstruktiv, transaktiv	Prinzipien der Pathogenese und Pathomechanismen<br>Diagnostische Verfahren	Medizinischer Experte<br>Kommunikator	Verstehen, Anwenden, Analysieren
17:45-18:00	Tagesevaluation und Abschluss	Evaluationsbogen					

Abkürzungen: ICAP, *interactive, constructive, active, passive*; OI, *Osteogenesis imperfecta*

Zusätzliche Tab. 3: Ablauf Präsenzphase II. Ablauf Präsenzphase I. Dargestellt sind die Zeiten inkl. der Dauer der einzelnen Schritte, die Themen, didaktische Methoden, Ziel der jeweiligen didaktischen Methoden, Kompetenzen sowie Kompetenz- und Arztrollen nach NKLM und die Kompetenzebenen nach Bloom, 1984.

Zeit	Thema	Didaktische Methode	Ziel der didaktischen Methode	ICAP	Kompetenz / Medizinisches Wissen	Kompetenz- / Arztrollen	Kompetenzebenen (Bloom, 1984)
14:00-14:15	Begrüßung, Evaluation, Vorschau	Evaluationsfragen Filme<br>Agenda / Tageslernziele (Flipcharts an Wand)	Übersicht des Tagesablauf				
14:15-14:45	Ursache, Schweregrad, Diagnostik und Therapie von OI (anhand eines klinischen Falls)	Interaktiver Vortrag (Power Point mit integrierten Aufgaben und Diskussion)	Informationsvermittlung mit Aktivierung<br>Anwenden, vertiefen der biochemischen Grundlagen	passiv, aktiv, konstruktiv, transaktiv	Prinzipien der Pathogenese und Pathomechanismen<br>Diagnostische Verfahren<br>Therapeutische Przinzipien	Medizinischer Experte<br>Kommunikator	Verstehen, Anwenden, Analysieren
14:45-15:15	Ehlers-Danlos-Syndrom / Skorbut (Biochem. Hintergrund, Klassifizierung und Symptome)	Impulsvortrag (Power Point)	Informationsvermittlung mit Aktivierung	passiv	Prinzipien der Pathogenese und Pathomechanismen	Medizinischer Experte	Verstehen
15:15-15:20	Erläuterung der Gruppenarbeit	Aufgaben mit Szenarien aus späterem Berufsleben					
15:20-15:40	Gruppe 1 / 2: OI (Experte / Laie)<br>Gruppe 3 / 4: EDS (Experte / Laie)<br>Gruppe 5 / 6: Skorbut (Experte / Laie)	Gruppenarbeit<br>Diskussion	Aktivieren und Vertiefen des gesamten Stoffes	konstruktiv	Prinzipien der Pathogenese und Pathomechanismen	Medizinischer Experte<br>Mitglied im Team<br>Kommunikator	Anwenden
15:40-15:55	Pause						
15:55-16:40	Gegenseitige Erläuterung:<br>Gruppe 1+2: OI (Experte/Laie)<br>Gruppe 3+4: EDS (Experte/Laie)<br>Gruppe 5+6: Skorbut (Experte/Laie)	Gruppenarbeit<br>Erläuterung und Diskussion<br>Wahl des Rollenspiels<br>Interne Aufgabenverteilung	Anwenden und vertiefen des gesamten Stoffes	transaktiv	Prinzipien normaler Struktur und Funktion<br>Prinzipien der Pathogenese und Pathomechanismen	Medizinischer Experte<br>Kommunikator<br>Ärztliche Gesprächs-führung	Analysieren
16:40-16:40	Vorstellung der Gruppenarbeit (3 aus 6 Themen	Rollenspiele vor Plenum: Gespräch zwischen Arzt (Studierende) und Experte / Laie (Dozenierende)<br><br>Zuhörer: Fragebogen<br><br>Diskussion im Plenum	Anwenden und vertiefen des gesamten Stoffes	transaktiv	Prinzipien normaler Struktur und Funktion<br>Prinzipien der Pathogenese und Pathomechanismen	Medizinischer Experte<br>Kommunikator<br>Ärztliche Gesprächs-führung	Analysieren
17:40-18:00	Evaluation<br>Wissenstest<br>Abschluss	Evaluationsfragen Gesamt-veranstaltung<br>Lernkontrolle	Feedback<br>Feststellung des Wissenserwerb	aktiv			

Abkürzung: EDS, Ehlers-Danlos-Syndrom; ICAP, *interactive, constructive, active, passive*; OI, *Osteogenesis imperfecta*

5 Exemplarische Aufgaben der Präsenzphasen I und II

5.1 Exemplarische Aufgabe der Präsenzphase I

Arbeitsauftrag „Prüfungssimulation“, Prüfung 1

Arbeitsgruppenmitglieder:

....

Sie befinden sich in der mündlichen M1 Prüfung. Ihr Biochemie Prüfer stellt Ihnen die Aufgabe, die Transkription inklusive deren Regulation zu erläutern. Papier und Stifte liegen vor. Bitte versuchen Sie heute, diese Thematik auf einem Flipchart zu skizzieren. Im Anschluss wird einer aus der Gruppe die Gelegenheit bekommen, das Thema anhand des Flipcharts in ca. 5 Minuten wie in einer Prüfung vorzustellen.

Inhalte in Stichpunkten:

- Promotor mit cis-regulatorischen Elementen, TATA Box und Transkriptionsstartpunkt
- Transkriptionsfaktoren, RNA-Polymerase
- Transkription mit RNA-Polymerase, Matrizen und Template DNA-Strang, hnRNA, Lese- und Syntheserichtung, Nukleotide

Phase 1: Die Vorbereitung

- Bitte überlegen Sie in der Gruppe, wie Sie diese Aufgabe in einer Prüfung lösen würden. Bereiten Sie ein Flipchart mit den wichtigsten Fakten und Prozessen vor. Beachten Sie dabei folgendes:
  - Geben Sie eine Übersicht der Inhalte (z.B. Wozu dient der Prozess? Was passiert in diesem?).
  - Kommen Sie dann vom Großen zum Kleinen (z.B. Was wird dafür benötigt? Wie verläuft der Prozess?).
  - Visualisieren Sie die wichtigsten Fakten und komplizierten Prozesse.
  - Erläutern Sie nur wenige Inhalte, die Sie nicht visualisieren.
  - Beschreiben Sie alle Inhalte, ob visualisiert oder nicht, nachvollziehbar.
  - Sprechen Sie möglichst frei (kein Ablesen).

- Wählen Sie einen aus der Gruppe, der die Rolle des „Prüflings“ in der Prüfungssimulation übernimmt.
- Dauer: 30 min

Phase 2: Die Prüfungssimulation

- Eine Person aus der Gruppe wird in die Rolle des Prüflings schlüpfen.
- Ihre Dozentin wird die Biochemie-Prüferin darstellen und als Ausgangsfrage oben genannte Frage stellen.
- Versuchen Sie, die Antwort auf diese relativ offene Frage strukturiert darzulegen.

- Seien Sie darauf eingestellt, das – wie in einer realen Prüfung – Zwischenfragen gestellt werden können.

5.2 Exemplarische Aufgaben der Präsenzphase II

Arbeitsauftrag „Expertengruppe“ *Osteogenesis imperfecta*

„Expertengruppe“:
.....

Tandemgruppe:
.....

Klinisches-wissenschaftliches Szenario:

Sie befinden sich auf einem wissenschaftlichen Kongress und stellen in einem Fachvortrag Ihre neuesten Daten auf dem Gebiet der *Osteogenesis imperfecta* Forschung dar. Sie konnten eine bis dato unbekannte Mutation im *COL1A1* Gen (im N-Terminus der Kollagen-α-Kette) identifizieren, welche *Osteogenesis imperfecta* Typ IV auslöst. Im Anschluss an Ihren Vortrag kommt eine Kollegin auf Sie zu, der sich von Ihnen den allgemeinen biochemischen Hintergrund von *Osteogenesis imperfecta* erläutern lässt.

Phase 1: Die Vorbereitung
- Bitte diskutieren Sie in den nächsten 25 Minuten in Ihrer Kleingruppe, wie Sie dabei vorgehen würden.
- Im Anschluss treffen Sie sich mit Ihrer Tandemgruppe.

Phase 2: Die Vorstellung vor der Tandemgruppe
- Stellen Sie Ihrer Tandemgruppe das Szenario vor.
- Einer von Ihrer Gruppe darf nun in die Rolle des Forschers schlüpfen und in ca. 5-10 Minuten vor Ihrer Tandemgruppe die Erläuterung des Experten vornehmen.
- Ihre Tandemgruppe wiederum simuliert in ebenfalls 5-10 Minuten deren Szenario.
- Bitte entscheiden Sie, welche der beiden Szenarien Sie im Anschluss an der Tafel simulieren wollen.
- Wählen Sie zwei aus der Gruppe, die verschiedenen Rollen übernehmen.

Phase 3: Die Simulation
- Eine Person aus der Gruppe wird in die Rolle des Forschers oder Kinderarztes schlüpfen.
- Eine zweite Person wird die Rolle der Kollegin oder der Mutter übernehmen.
- Dauer: 5 Minuten

Beachten Sie bei der Simulation folgendes:
- Geben Sie eine Übersicht der Inhalte (z.B. Wozu dient der Prozess? Was passiert dabei?).

- Kommen Sie dann vom Großen zum Kleinen (z.B. Was wird benötigt? Wie verläuft der Prozess?).
- Visualisieren Sie die wichtigsten Fakten und komplizierten Prozesse.
- Erläutern Sie nur wenige Inhalte, die Sie nicht visualisieren.
- Beschreiben Sie alle Inhalte, ob visualisiert oder nicht, nachvollziehbar.
- Sprechen Sie möglichst frei (kein Ablesen).
- Überlegen Sie sich außerdem, was die Kollegin oder Mutter konkret fragen könnte. Seien Sie kreativ, aber bleiben Sie realistisch.

Arbeitsauftrag „Laiengruppe" *Osteogenesis imperfecta*

„Laiengruppe":
....

Tandemgruppe:
....

Klinisches Szenario:

Sie sind Kinderarzt. Eine Mutter sucht Sie mit ihrer 5-jährigen Tochter aufgrund eines fiebrigen Infekts auf. Ihnen fällt auf, dass das Kind einen Gips am rechten Unterarm trägt. Sie erinnern sich, dass das Kind bei einem der letzten Besuche ebenfalls einen Gips getragen hat, damals aber am rechten Bein. Bei Nachfrage bestätigt Ihnen das die Mutter und erzählt außerdem, dass das Mädchen in den letzten zwei Jahren insgesamt fünf Knochenbrüche erlitten hat. Weitere Untersuchungen zeigen, dass das Kind vermutlich nicht misshandelt wurde. Ihre Verdachtsdiagnose lautet *Osteogenesis imperfecta Typ I*, welche durch Ihre Untersuchungen bestätigt wird. Sie klären die Mutter über die Krankheit auf, schildern deren biochemischen Hintergrund anhand einer einfachen Skizze, wobei Sie auch die Ursache für die Unheilbarkeit erläutern.

Phase 1: Die Vorbereitung
- Bitte diskutieren Sie in den nächsten 25 Minuten in Ihrer Kleingruppe, wie Sie dabei vorgehen würden.
- Im Anschluss treffen Sie sich mit Ihrer Tandemgruppe.

Phase 2: Die Vorstellung vor der Tandemgruppe
- Stellen Sie Ihrer Tandemgruppe das Szenario vor.
- Einer von Ihrer Gruppe darf nun in die Rolle des Kinderarztes schlüpfen und in ca. 5-10 Minuten vor Ihrer Tandemgruppe die Erläuterung des Arztes vornehmen.
- Ihre Tandemgruppe wiederum simuliert in ebenfalls 5-10 Minuten deren Szenario.
- Bitte entscheiden Sie, welche der beiden Szenarien Sie im Anschluss an der Tafel simulieren wollen.
- Wählen Sie zwei aus der Gruppe, die verschiedenen Rollen übernehmen.

Phase 3: Die Simulation

- Eine Person aus der Gruppe wird in die Rolle des Forschers oder Kinderarztes schlüpfen.
- Eine zweite Person wird die Rolle der Kollegin oder der Mutter übernehmen.
- Dauer: 5 Minuten

Beachten Sie bei der Simulation folgendes:

- Geben Sie eine Übersicht der Inhalte (z.B. Wozu dient der Prozess? Was passiert dabei?).
- Kommen Sie dann vom Großen zum Kleinen (z.B. Was wird benötigt? Wie verläuft der Prozess?).
- Visualisieren Sie die wichtigsten Fakten und komplizierten Prozesse. Im Laiengespräch: Nehmen Sie Bezüge zu Alltäglichem.
- Erläutern Sie nur wenige Inhalte, die Sie nicht visualisieren.
- Beschreiben Sie alle Inhalte, ob visualisiert oder nicht, nachvollziehbar.
- Sprechen Sie möglichst frei (kein Ablesen).
- Überlegen Sie sich außerdem, was die Kollegin oder Mutter konkret fragen könnte. Seien Sie kreativ, aber bleiben Sie realistisch.

6 Wissenstest Präsenzphase O und II

Aufgabe 1

Bitte markieren Sie die (eine) korrekte Aussage in der rechten Spalte mit einem „X“.

	Bei der Regulation der Transkription.....	
**A**	können die Transkriptionsfaktoren sowohl hemmend als auch aktivierend wirken.	
**B**	spielen die Faktoren eIF2 und eIF4 eine wichtige Rolle.	
**C**	ist das Startkodon ATG beteiligt.	
**D**	wirken die cis-regulatorischen Enhancer Elemente hemmend.	
**E**	hat die RNA-Polymerase keine Funktion.	
Punkte (von der Dozentin auszufüllen)		

Max.1 Punkt

Aufgabe 2

*Bitte markieren Sie die (eine) Aussage, die **NICHT** korrekt ist, in der rechten Spalte mit einem „X“.*

**A**	SRP steht für *signal recognition particle*.	
**B**	Chaperone unterstützen die Proteinfaltung im rER.	
**C**	Die Proteindisulfid-Isomerase sorgt für die korrekte Bildung von Disulfidbrücken im Protein.	
**D**	Proteine werden an Asparaginreste O-glykosidisch mit Kohlenhydrateinheiten modifiziert.	
**E**	Der Golgi-Apparat wird in cis, medianer und trans Golgi unterteilt.	
Punkte (von der Dozentin auszufüllen)		

Max.1 Punkt

Aufgabe 3

*Bitte entscheiden Sie für **JEDE** Aussage, ob sie korrekt ist oder nicht. Markieren Sie dies entsprechend in der rechten Spalte mit einem „X“.*

Bei der Kollagensynthese.....	korrekt	nicht korrekt
werden Kohlenhydrate über eine N-glykosidische Bindung an einen Hydroxylysylrest angehängt.		
spielen für die Tripelhelixbildung Disulfidbrücken am N-Propeptid eine entscheidende Rolle.		
katalysieren Peptidasen eine Abspaltung der Propeptide, wodurch Tropokollagen entsteht.		
katalysiert die Lysyloxidase die Umwandlung von Hydroxylysin zu Hydroxyallysin.		
Punkte (von der Dozentin auszufüllen)		

Max. 4 Punkte

Aufgabe 4

Klin. Fallvignette:

Sie sind Kinderarzt. Eine Mutter sucht Sie mit ihrer 5-jährigen Tochter aufgrund eines fiebrigen Infekts auf. Ihnen fällt auf, dass das Kind einen Gips am rechten Unterarm trägt. Sie erinnern sich, dass das Kind bei einem der letzten Besuche ebenfalls einen Gips getragen hat, damals aber am rechten Bein. Bei Nachfrage bestätigt Ihnen das die Mutter und erzählt außerdem, dass das Mädchen in den letzten zwei Jahren insgesamt fünf Knochenbrüche erlitten hat. Weitere Untersuchungen zeigen, dass das Kind vermutlich nicht misshandelt wurde.

Frage 1:

Ihre Verdachtsdiagnose lautet *Osteogenesis imperfecta*. Welche Untersuchungen führen Sie durch, um diese Diagnose zu bestätigen? *Bitte markieren Sie in der rechten Spalte jene Untersuchungen, die Sie durchführen sollten, mit einem „X".*

**A**	Röntgen des Skeletts	
**B**	MRT der Leber	
**C**	Augenärztliche Untersuchungen	
**D**	Echokardiographie des Herzens	
**E**	Erhebung des Zahnstatus	
Punkte (von der Dozentin auszufüllen)		

Max. 3 Punkte

Frage 2:

Ihre ersten Untersuchungen bestätigen, dass das Mädchen an *Osteogenesis imperfecta* leidet. Um diese Diagnose zu sichern und zu klassifizieren, veranlassen Sie eine DNA-Sequenzierung. Welche Gene sollten Sie sequenzieren lassen? *Bitte markieren Sie in der rechten Spalte jene Gene, die Sie sequenzieren lassen sollten, mit einem „X".*

**A**	*Elastin Typ 1*	
**B**	*Collagen Typ 1 alpha 2*	
**C**	*Collagen Typ 10 alpha 1*	
**D**	*Collagen Typ 1 alpha 1*	
Punkte (von der Dozentin auszufüllen)		

Max. 2 Punkte

Frage 3:

Die DNA-Sequenzierung ergibt, dass das Kind an *Osteogenesis imperfecta* des Typs I leidet und die Mutter muss darüber informiert werden, dass diese Erkrankung derzeit noch unheilbar ist. Was kann eine biochemische Begründung für die Unheilbarkeit der Erkrankung sein? *Bitte markieren Sie die korrekte Aussage in der rechten Spalte mit einem „X".*

**A**	*Osteogenesis imperfecta* basiert auf einer aktivierende Mutation im Gen für die Lysylhydroxylase.	
**B**	*Osteogenesis imperfecta* wird durch einen Medikamentenmissbrauch induziert.	
**C**	*Osteogenesis imperfecta* basiert auf Mutationen im *Collagen Typ 1 apha 1* Gen.	
**D**	*Osteogenesis imperfecta* beruht auf einer Mangelernährung.	
Punkte (von der Dozentin auszufüllen)		

Max. 1 Punkte

7 Evaluationsbögen

7.1 Offizieller Evaluationsbogen der Medizinischen Fakultät Ulm

Gezeigt sind nur jene Items, die in dieser Studie ausgewertet wurden. Der Fragebogen wurde online über Moodle für die Studierenden beider Gruppen (traditionell und Inverted Classroom) bereitgestellt.

[SURVEY]

[ORGANIZATION]	[SUBUNIT]
[AUTHOR]	[PERIOD]

Markierung: ☐ ☒ ☐ ☐ ☐ Bitte verwenden Sie einen Kugelschreiber oder einen nicht zu starken Filzstift. Dieser Fragebogen wird maschinell erfasst.
Korrektur: ☐ ■ ☐ ☒ ☐ Bitte beachten Sie im Interesse einer optimalen Datenerfassung die links gegebenen Hinweise beim Ausfüllen.

Liebe Studentinnen und Studenten,
bitte schätzen Sie anhand dieses Bogens die von Ihnen besuchte Lehrveranstaltung ein. Die Ergebnisse werden in anonymisierter Form ausgewertet und den Dozierenden zurückgemeldet. Mit Ihrer sorgfältigen Beantwortung der Fragen unterstützen Sie die Bemühungen um eine effektive Lehre an der Medizinischen Fakultät Ulm.
Vielen Dank für Ihre Mithilfe.

Organisation, Struktur und Aufbau der Lehrveranstaltung

Item									
Die Lehrveranstaltung ist gut organisiert.	trifft gar nicht zu	☐	☐	☐	☐	☐	☐ trifft völlig zu	Frage ist nicht relevant	☐
Die Veranstaltung hat eine gute Gesamtstruktur ("Roter Faden").	trifft gar nicht zu	☐	☐	☐	☐	☐	☐ trifft völlig zu	Frage ist nicht relevant	☐
Es gab keine schweren Verspätungen oder Ausfälle.	trifft gar nicht zu	☐	☐	☐	☐	☐	☐ trifft völlig zu	Frage ist nicht relevant	☐

Lehrengagement der an der Lehrveranstaltung beteiligten Dozierenden

Item									
Den beteiligten Lehrenden ist der Lernerfolg der Studierenden wichtig.	trifft gar nicht zu	☐	☐	☐	☐	☐	☐ trifft völlig zu	Frage ist nicht relevant	☐
Die beteiligten Lehrenden sind gut auf den Unterricht vorbereitet.	trifft gar nicht zu	☐	☐	☐	☐	☐	☐ trifft völlig zu	Frage ist nicht relevant	☐
Die Studierenden werden motiviert, sich aktiv mit den Lerninhalten auseinanderzusetzen.	trifft gar nicht zu	☐	☐	☐	☐	☐	☐ trifft völlig zu	Frage ist nicht relevant	☐

Lernziele und Lerninhalte der Lehrveranstaltung

Item									
Die gestellten Anforderungen sind adäquat.	trifft gar nicht zu	☐	☐	☐	☐	☐	☐ trifft völlig zu	Frage ist nicht relevant	☐

Die Lernziele der Lehrveranstaltung sind transparent.	trifft gar nicht zu ☐	☐	☐	☐	☐	☐ trifft völlig zu	Frage ist nicht relevant ☐
Die Relevanz der Lerninhalte wird deutlich.	trifft gar nicht zu ☐	☐	☐	☐	☐	☐ trifft völlig zu	Frage ist nicht relevant ☐
Ich habe in der Lehrveranstaltung viel gelernt.	trifft gar nicht zu ☐	☐	☐	☐	☐	☐ trifft völlig zu	Frage ist nicht relevant ☐

Didaktische Umsetzung

Die beteiligten Lehrenden können die Lehrveranstaltungsinhalte gut vermitteln.	trifft gar nicht zu ☐	☐	☐	☐	☐	☐ trifft völlig zu	Frage ist nicht relevant ☐
Die beteiligten Lehrenden gestalten den Unterricht anschaulich (mit praktischen Bezügen, Beispielen, etc.).	trifft gar nicht zu ☐	☐	☐	☐	☐	☐ trifft völlig zu	Frage ist nicht relevant ☐
Die begleitenden Unterrichtsmaterialien (Skripte, Präsentationen, etc.) sind lernförderlich.	trifft gar nicht zu ☐	☐	☐	☐	☐	☐ trifft völlig zu	Frage ist nicht relevant ☐

Gesamtbewertung der Lehrveranstaltung

Wenn man alles in einer Note zusammenfassen könnte, würde ich der Veranstaltung die folgende Note (Schulnote von 1 bis 6) geben: ☐ **1** ☐ **2** ☐ **3** ☐ **4** ☐ **5** ☐ **6**

Zu optimieren wäre an der Veranstaltung (Mehrfachnennungen sind möglich):

- ☐ es besteht kein Handlungsbedarf
- ☐ Organisation, Struktur und Aufbau
- ☐ Methodik und Didaktik
- ☐ Veranstaltungsinhalte
- ☐ Sonstiges

Lob, Kritik und Verbesserungsvorschläge

Im Folgenden haben Sie die Möglichkeit, konkrete Verbesserungsvorschläge zu nennen (ggf. Bezug zu einzelnen Dozierenden nehmen).

Im Folgenden haben Sie die Möglichkeit, Lob zu äußern (ggf. Bezug zu einzelnen Dozierenden nehmen).

7.2 Evaluation zur Gesamtveranstaltung Integriertes Seminar Modul 6 – Pathobiochemie II: „Vom Gen zum Protein“

Der Fragebogen wurde im Papierformat am Ende der Präsenzphase II den Inverted Classroom Gruppen bereitgestellt.

	Ja	Nein
Ich habe mich auf die Präsenzphase I vorbereitet.		
Ich habe mich auf die Präsenzphase II vorbereitet.		

1 = trifft **vollkommen** zu
5 = trifft **überhaupt nicht** zu

	1	2	3	4	5
Die Einführungsveranstaltung (Präsenzphase 0) hat mich zur Vorbereitung auf die Präsenzphasen I und II motiviert.					
Die Vorbereitungsmaterialien waren gut strukturiert.					
Die Vorbereitungsmaterialien waren vom Umfang angemessen.					
Ich hätte auch alle drei Lehrfilme vor Präsenzphase I angesehen.					
Die Vorbereitungsmaterialien haben mich zum Lernen motiviert.					
Die Vorbereitungs- und Präsenzphasen waren inhaltlich gut aufeinander abgestimmt.					
Die Veranstaltungsinhalte sind für mein Medizinstudium relevant.					
Insgesamt hat mich die Veranstaltung dazu motiviert, mich weiter mit den vermittelten Inhalten zu beschäftigen.					
Die Veranstaltung hat mein Interesse für die Biochemie gesteigert.					
Durch die Veranstaltung wurde ich gut auf kommende Prüfungen vorbereitet.					
Wenn ich die Veranstaltung mit anderen Seminaren vergleiche, dann habe ich durch den Einsatz der *Inverted Classroom* Methode zusätzliche Kompetenzen (wie z.B. Arbeiten im Team, ärztliche Kommunikation wie die Diskussion mit Fachkollegen oder das Erläutern von Sachverhalten gegenüber Fachfremden) trainiert.					
Durch die klinischen und wissenschaftlichen Szenarien wurde mir die Relevanz des biochemischen Themas deutlich.					

Wenn ich die Veranstaltung mit anderen vergleiche, dann muss ich durch den Einsatz der *Inverted Classroom* Methode weniger Zeit für Nachbearbeitung aufwenden.					

Insgesamt gebe ich der Veranstaltung (Vorbereitungs- und Präsenzphase) folgende Schulnote:

1 (sehr gut) 2 (gut) 3 (befriedigend) 4 (ausreichend) 5 (mangelhaft) 6 (ungenügend)

Hier haben Sie die Möglichkeit, Lob und Kritik zu äußern:

7.3 Evaluationen zu den drei Pilotlehrfilmen SS2016 (*Inverted Classroom* Gruppe)

Der Fragebogen wurde im Papierformat am Ende der Präsenzphase II den Inverted Classroom Gruppen bereitgestellt.

Ich habe die Lehrfilme angesehen.		
Die Lehrfilme waren als Vorbereitung auf die Präsenzzeit hilfreich.		
Ich würde mir diese Art der Lehrfilme begleitend gerne auch zu anderen Veranstaltungen (wie z.B. Biochemie Hauptvorlesung) der Biochemie ansehen.		

1 = trifft **vollkommen** zu
5 = trifft **überhaupt nicht** zu

	**1**	**2**	**3**	**4**	**5**
Die Lehrfilme haben meine Motivation für das Seminar erhöht.					
Die Lerninhalte waren gut strukturiert.					
Die Lehrfilme waren inhaltlich klar verständlich.					
Das inhaltliche Niveau war angemessen.					
Die Aufgaben zur Selbstlernkontrolle waren hilfreich, den Stoff zu durchdringen.					
Die Vorbereitungs- und Präsenzphasen waren inhaltlich gut aufeinander abgestimmt.					

Für die Lehrfilme gebe ich folgende Schulnote:

1 (sehr gut) 2 (gut) 3 (befriedigend) 4 (ausreichend) 5 (mangelhaft) 6 (ungenügend)

Hier haben Sie die Möglichkeit, Lob und Kritik zu äußern:

7.4 Evaluationen zu den drei Pilotlehrfilmen SS2016 (traditionelle Gruppe)

Der Fragebogen wurde online über Moodle für die Studierenden der traditionellen Gruppen bereitgestellt.

	Ja	Nein
Die Lehrfilme wären als Vorbereitung auf das Integrierte Seminar „Pathobiochemie II: Vom Gen zum Protein" hilfreich gewesen.		
Ich würde mir diese Art der Lehrfilme begleitend gerne auch zu anderen Veranstaltungen (wie z.B. Biochemie Hauptvorlesung) der Biochemie ansehen.		

1 = trifft **überhaupt nicht** zu
5 = trifft **vollkommen** zu

	1	2	3	4	5
Die Lehrfilme hätten meine Motivation für das Seminar erhöht.					
Die Lerninhalte waren gut strukturiert klar verständlich.					
Das inhaltliche Niveau war angemessen.					

Für die Lehrfilme gebe ich folgende Schulnote:

1 (sehr gut) 2 (gut) 3 (befriedigend) 4 (ausreichend) 5 (mangelhaft) 6 (ungenügend)

Hier haben Sie die Möglichkeit, Lob und Kritik zu äußern.
